# Sequential Regulation of Neurovascular Bone Regeneration in Large Bone Defects Using Black Phosphorus‐Europium Nanosheet Integrated TPMS Biomimetic Scaffold with Photo‐Controlled Ion Release

**DOI:** 10.1002/advs.77652

**Published:** 2026-09-16

**Authors:** Bingqian Wang, Yuping Zhang, Xiaoning Su, Ning Liu, Hui Zeng, Bo Yang, Yang Xue, Fang Guo, Changkui Liu

**Affiliations:** ^1^ Engineering Research Center Oral Biomaterials and Advanced Equipments Research Center of Dental and Maxillofacial Tissue Regeneration and Repair Technology School of Stomatology Xi'an Medical University Xi'an P. R. China; ^2^ State Key Laboratory of Oral and Maxillofacial Reconstruction and Regeneration National Clinical Research Center for Oral Diseases Shaanxi Clinical Research Center for Oral Diseases Department of Oral and Maxillofacial Surgery School of Stomatology The Fourth Military Medical University Xi'an P. R. China

**Keywords:** black phosphorus nanosheets, bone defect repair, europium, mild hyperthermia therapy, neuro‐vascularization, osteogenesis, triply periodic minimal surfaces

## Abstract

Titanium alloy scaffolds (Ti–6Al–4 V) are widely used to repair bone defects. However, conventional titanium‐based implants lack customized functional surface interfaces that promote coordinated local nerve and blood vessel regeneration, thereby limiting bone regeneration and long‐term stability. To address this limitation, we utilize a topologically optimized triple‐periodic minimal surface (TPMS) porous titanium (Ti) scaffold. Polydopamine (PDA) surface engineering is used to stably immobilize europium ions (Eu^3+^)‐coordinated black phosphorus (BP) nanosheets onto the surface, forming a Ti–PDA@(BP+Eu) (TPBE) scaffold with photothermal‐ionic synergistic release to coordinate early neurovascular reconstruction and subsequent bone regeneration. In vitro studies demonstrate that under periodic near‐infrared irradiation, the TPBE scaffold enables on‐demand release of Eu^3^^+^/phosphate ions (PO_4_^3−^), thereby promoting adhesion, migration, differentiation, and gene expression of neural, vascular, and osteogenic cells. Further, activating the PI3K/Akt pathways enhances neurite outgrowth and axonal regeneration of rat adrenal pheochromocytoma cells. In vivo large‐segment bone defect models demonstrate that TPBE scaffolds combined with mild photothermal therapy increases neurovascular network density and upregulate osteogenic–neuro–vascular coupling factors, thereby accelerating bone regeneration. This study presents an innovative method for the fabrication of Ti‐based implant materials capable of cascade regeneration of nerve, vascular, and bone tissues, establishing the basis for the exploration of multitissue regenerative biomaterials.

## Graphical abstract

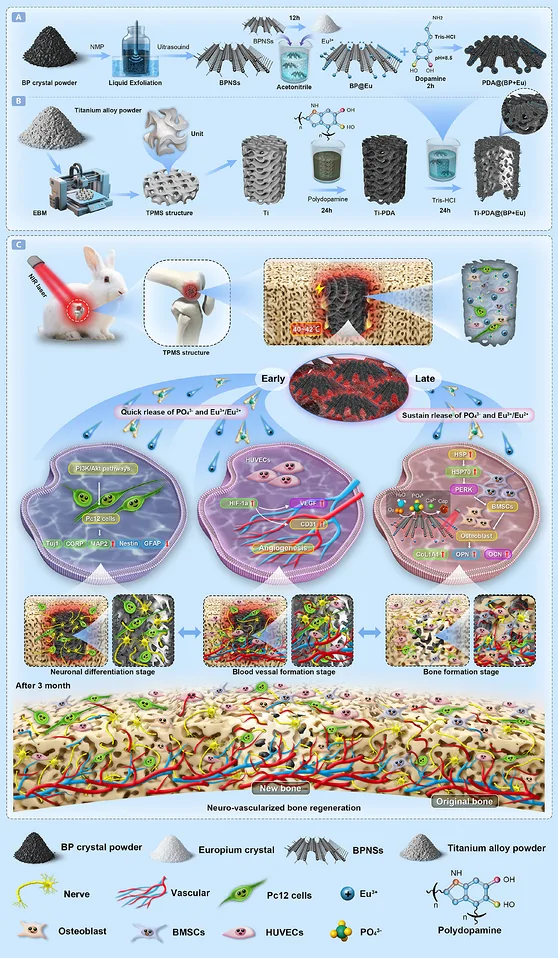

## 1 Introduction

Repairing large‐segment bone defects resulting from trauma, infection, bone diseases, or surgical excision remains a major challenge in bone regeneration research [1]. Each year, nearly 15 million bone injuries occur worldwide, causing a continuous pressure on medical services [2]. Autologous and allogeneic bone grafts, which exhibit good porosity and mechanical matching performance, remain the primary methods for repairing bone defects. However, their widespread application is limited by several drawbacks, including insufficient donor supply, surgical complications, immune response, and infection risks [3]. Recently, porous titanium alloy (Ti–6Al–4 V) scaffolds, optimized using triple‐periodic minimal surface (TPMS) topology, have emerged as promising alternatives for bone defect repair. These scaffolds leverage the biomimetic mechanics of trabecular bone and promote cell migration and material transport [4, 5]. However, skeletal regeneration is a highly coordinated process involving neural, vascular, and osteogenic activities. Early and adequate neurovascularization improves the local microenvironment and directly regulates osteogenesis through neural‐bone interactions, thereby ensuring durable implant stability [6, 7, 8, 9]. However, TPMS porous titanium scaffolds generally lack bioactive surface interfaces that induce neural and vascular regeneration, thereby limiting their postimplantation regeneration efficiency and long‐term host integration [10, 11]. Therefore, in addition to optimizing scaffold topology and enhancing osteogenic capacity, incorporating a functional interface that actively promotes the early, coordinated regeneration of nerves and blood vessels into the implant is crucial for the design of materials intended for the repair of large bone defects.

Black phosphorus nanosheets (BPNSs), an emerging 2D nanomaterial, exhibit potential for bone tissue engineering owing to their high specific surface area, excellent biocompatibility, and degradability [12]. During degradation, released phosphate ions (PO_4_^3^^−^) capture calcium ion (Ca^2+^) to promote calcium phosphate (Ca_3_(PO_4_)_2_) deposition, thereby accelerating cell‐mediated biomineralization [13]. Further, BPNSs exhibit excellent near‐infrared (NIR) photothermal conversion, enabling precise and mild photothermal therapy that supports a favorable microenvironment for neurovascularized bone regeneration [14, 15]. However, their inherent instability and susceptibility to oxidation limit photothermal stability and potential biomedical applications [16, 17]. Metal‐ion coordination enhances the stability and bioactivity of BPNSs. Among the potential modifiers, trivalent europium ions (Eu^3^^+^) are promising owing to their low toxicity, calcium‐mimicking characteristics, and multifunctional roles in promoting neurovascularization and osteogenesis [18, 19, 20, 21].

This study presents a P–Eu coordination strategy for enhancing the structural stability and biological functionality of BPNSs. However, achieving stable immobilization of Eu^3^^+^‐modified black phosphorus (BP) nanocomposites (BP+Eu) on titanium implant surfaces while enabling time‐controlled Eu^3^^+^ release during bone repair remains a critical challenge. Polydopamine (PDA), a biomimetic adhesive coating, readily forms stable layers on diverse substrates and immobilizes nanomaterials through catechol‐mediated interfacial bonding [22, 23]. Furthermore, PDA coatings improve the environmental stability of BPNSs while enhancing their photothermal and biological activity [24].

**FIGURE 1:**
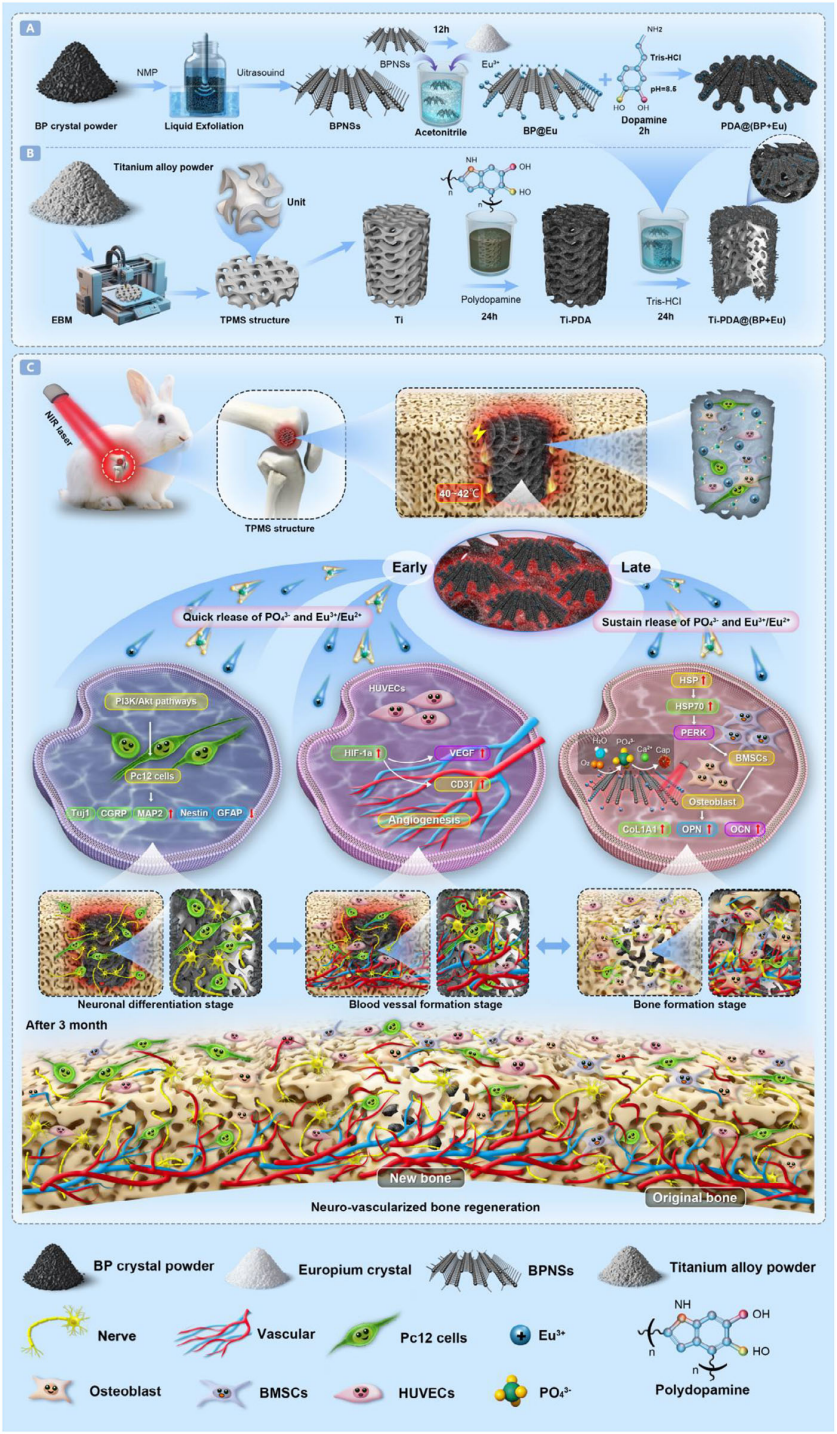
Schematic of the preparation and application of Ti–PDA@(BP+Eu) scaffolds: (A,B) Fabrication of PDA@(BP+Eu) nanosheets and Ti–PDA@(BP+Eu) scaffolds; (C) Ti–PDA@(BP+Eu) scaffold, combined with mild photothermal therapy, promotes rabbit femoral condyle defect repair by enabling early neurovascularization and sustained osteogenesis.

This study presents a multifunctional composite coating system (Ti–PDA@(BP+Eu)) based on PDA interfacial engineering. Electron beam melting (EBM) is employed to fabricate porous titanium scaffolds with a TPMS structure. The excellent adhesion and active sites of PDA are leveraged to stably immobilize P–Eu‐coordinated modified BPNSs (PDA@(BP+Eu)) onto the scaffold surface, forming a light‐responsive bone‐repairing Ti–PDA@(BP+Eu) scaffold. This scaffold enhances BPNSs stability and enables the sequential and photothermally regulated release of Eu^3^^+^/PO_4_^3−^ ions. This promotes early neurovascular network formation and osteogenic differentiation, thereby accelerating bone tissue regeneration and repair (Figure 1). This approach offers a valuable platform for designing load‐bearing biomaterials, thereby enhancing the reconstruction of neuro–vascular–bone networks and improving the repair efficacy of bone defects.

## 2 Materials and Methods

### 2.1 Reagents and Chemicals

BP crystal powder (101127) was purchased from Nanjing/Jiangsu XFNANO Materials Tech Co., Ltd.; Eu(NO_3_)_3_·6H_2_O (A43301) was obtained from Energy Chemical Co., Ltd. (Shanghai, China); tris‐hydrochloride buffer (Tris‐HCl), *N*‐methylpyrrolidone (1‐methyl‐2‐pyrrolidinone, NMP), dopamine hydrochloride, acetone, and ethanol were purchased from Aladdin Biochemical Technology Co., Ltd. (Shanghai, China). Titanium scaffolds were provided by the Northwest Institute of Nonferrous Metals Research (Xi'an, China).

### 2.2 Preparation and Characterization of PDA@(BP+Eu) Nanosheets

#### 2.2.1 Preparation of BPNSs

Two‐dimensional BPNSs were prepared using liquid‐phase exfoliation. BP crystals were stored in a dark argon glovebox before use to prevent oxidation. First, 50 mg of bulk BP powder was dispersed in 100 mL of NMP. The dispersion was then sonicated using a probe sonicator (450 W, on/off cycle: 5 s/5 s) in an ice bath for 12 h under an inert atmosphere. The resulting suspension was centrifuged at 15 000 rpm for 20 min to remove the unexfoliated particles. The supernatant containing BPNSs was collected and stored at 4°C for further use.

#### 2.2.2 PDA@(BP+Eu) Nanosheet Encapsulation

PDA@(BP+Eu) nanosheets were synthesized by employing a stepwise approach. First, Eu(NO_3_)_3_·6H_2_O and 500 µg/mL BPNSs at varied mass ratios were co‐incubated in an acetonitrile solution for 12 h. Centrifugation (4°C, 12 000 r/min, and 15 min) and washing yielded numerous BP+Eu precursors. The products were placed in a 10% hydrogen peroxide (H_2_O_2_) solution and fully oxidized at 50°C for 48 h. The concentrations of phosphorus (P) and europium (Eu) were then measured using inductively coupled plasma optical emission spectroscopy (ICP‐OES; PerkinElmer, Optima 7000DV, USA), and the maximum Eu^3+^ loading capacity on BPNSs was calculated. In addition, 2 mg of BP+Eu nanosheets at the target loading ratio was dispersed in 2 mg/mL PDA (pH = 8.5) and subjected to in situ polymerization for 2 h. The final product was obtained through centrifugation, washing, and drying. PDA@BP nanosheets prepared without Eu^3^^+^ served as controls. All samples were stored at 4°C in the dark.

#### 2.2.3 Characterization of BPNSs and PDA@(BP+Eu) Nanosheets

Surface morphology and thickness of BPNSs were characterized by performing scanning electron microscopy (SEM; JEOL, JSM‐IT200, Japan) and atomic force microscopy (AFM; Bruker Dimension ICON, Germany). Microstructure and elemental distribution of the materials were analyzed using transmission electron microscopy (TEM; JEM‐2100Plus) combined with energy‐dispersive spectroscopy (EDS; Oxford Xplore30, Oxford, UK). Nanoparticle size and zeta potential were measured using dynamic light scattering (Zetasizer Nano ZS90, Malvern Panalytical, UK). Phase structure was examined using Raman spectroscopy (LabRAM HR800, Horiba, Jobin Yvon, France). To evaluate photothermal performance, the temperature increase was measured under NIR irradiation (LWIRL808‐5 W, Beijing, China) for BPNSs dispersions at varying concentrations (25–100 µg/mL and 1 mL) and different sample formulations (phosphate‐buffered saline (PBS), BPNSs, BP+Eu, and PDA@(BP+Eu) at 50 µg/mL, 1 mL). All samples were irradiated at 808 nm and 1.0 W/cm^2^ for 10 min, with temperatures recorded every minute. Thermal distribution across different sample dispersions was monitored using an infrared thermal imager (Fluke TiS55, USA). The PDA@(BP+Eu) nanosheet dispersion was subjected to five cycles of 10‐min irradiation followed by 10‐min cooling to assess photothermal stability. Ultraviolet (UV)–visible spectrophotometry (TU‐1810, Beijing, China) was employed to measure the absorption spectra of BPNSs, BP+Eu, and PDA@(BP+Eu) dispersions at 50 µg/mL. Further, color changes during irradiation were recorded using a digital camera (D850, Nikon, China) for auxiliary analyses.

### 2.3 Fabrication and Characterization of 3D‐Printed Scaffolds

#### 2.3.1 Fabrication of Ti–PDA@(BP+Eu) Scaffolds

Porous titanium alloy scaffolds were fabricated using EBM technology [25]. The computer‐aided design file of a porous titanium alloy scaffold (wall thickness = 0.5 mm, pore diameter = 600 µm, and porosity = 60%) was imported into the EBM system (Arcam A1, Arcam AB, Molndal, Sweden). The scaffolds were printed layer by layer at an electron beam current of 6 mA and a speed of 15 000 mm/s and then mechanically polished. The scaffolds were then immersed in the PDA solution (2 mg/mL, pH = 8.5, 1 m Tris‐HCl) and incubated for 24 h in a shaking incubator (37°C, 100 rpm/min) in the dark to deposit a uniform PDA coating onto the scaffold surface. Ti–PDA intermediates were obtained after rinsing with deionized water and drying. Ti–PDA scaffolds were placed in dispersions of 50 µg/mL PDA@BP or PDA@(BP+Eu) nanosheets and reacted under similar conditions for 24 h. After thorough washing and vacuum drying, Ti–PDA@BP and Ti–PDA@(BP+Eu) composite scaffolds were obtained and stored at 4°C in the dark. The scaffolds are denoted as follows: T (pure titanium), TP (Ti–PDA), TPB (Ti–PDA@BP), TPBE (Ti–PDA@(BP+Eu)), and TPBE+NIR (TPBE under NIR irradiation).

#### 2.3.2 Characterization of TPBE Scaffolds

Compression tests were conducted on cylindrical scaffolds (*Φ*10×15 mm) using an electro‐hydraulic servo testing machine (SANS CMT4304, MTS Corporation, USA) according to ISO 13314:2011. Surface morphology and elemental distribution were characterized using SEM combined with EDS. Surface chemical composition was analyzed using X‐ray photoelectron spectroscopy (XPS; Shimadzu Kratos AXIS Ultra DLD, Japan). To evaluate the photothermal performance of TPBE scaffolds, T and TPBE were immersed in 1 mL PBS and irradiated using NIR light at a similar power for 10 min, and the final temperature change was recorded. Further, the TPBE scaffold underwent a 10‐min cyclic stability photothermal stability test as per the procedure described in Section 2.3.1. To investigate the effects of ^6^ ^0^Co sterilization and five photothermal cycles on the stability of the PDA@(BP+Eu) coating on the scaffold surface, SEM–EDS and Raman spectroscopy were employed to characterize the surface morphology, elemental distribution, and structural properties of the scaffolds before and after treatment. Thermal imaging systems were used to record the maximum surface temperatures of each sample group under NIR irradiation for 10 min, following the same methodology described earlier. Surface hydrophilicity/hydrophobicity was evaluated using contact angle measurements (SL200 A/B/D, Solon Tech, China), whereas the surface roughness was characterized using AFM. The scaffolds, prepared according to GB/T 16886.12‐200, were placed in PBS (pH = 7.4) and deionized water at a mass‐to‐volume ratio of 1 g:10 mL. Samples were collected at 1, 4, 8, 16, and 32 days. Eu^3^^+^ release in PBS was measured using ICP‐OES, whereas PO_4_^3^^−^ release in deionized water was quantified using a phosphate assay kit (E‐BC‐K832‐M, Elabscience). Fresh solutions were replenished after each sampling to maintain liquid volume constant.

### 2.4 In Vitro Experiments

#### 2.4.1 Cell‐Scaffold Coculture

Rat bone marrow mesenchymal stem cells (rBMSCs) were isolated from 3–4 week‐old Sprague Dawley rats. Animal experiments were approved by the Teaching and Research Ethics Committee. Cells were obtained by exposing the femoral shaft and flushing the medullary cavity. The isolated cells were cultured in Dulbecco's modified Eagle medium (DMEM)/F12 medium (Gibco, USA) supplemented with 10% fetal bovine serum (FBS; Sigma Aldrich, USA) and 1% penicillin/streptomycin (P/S; Beyotime, China) at 37°C in a 5% CO_2_ incubator (MCO‐15AC). Only passages 2–5 were used for experiments. Human umbilical vein endothelial cells (HUVECs) and rat adrenal pheochromocytoma cells (PC12) were obtained from the Chinese Academy of Sciences Cell Bank (Shanghai, China) and maintained and passaged under similar culture conditions in DMEM (Gibco, USA) supplemented with 10% FBS and 1% P/S. The isolation and culture of primary dorsal root ganglion (DRG) neurons from mice were performed according to previously reported methods [26]. Four‐week‐old C57 mice were used. The intervertebral foramina were exposed under a stereomicroscope, and bilateral DRG tissues were meticulously dissected and harvested and subsequently placed in a precooled Hank's balanced salt solution. The DRG tissues were minced and digested with a digestion buffer at 37°C for 35–40 min, followed by centrifugation at 1000 rpm for 5 min. The cells were resuspended in Neurobasal (A358290, Thermo Fisher Scientific) medium containing 2% B27 (A3582801, Thermo FisherScientific), 1% P/S, and 1% glutamine (25030081, Thermo Fisher Scientific). After 48 h of culture, 10 µm cytarabine was added to inhibit nonneuronal cell proliferation; this treatment was continued for another 48 h. Subsequently, the medium was replaced by half volume every three days, and the culture was maintained under standard conditions at 37°C and 5% CO_2_.

All scaffolds were sterilized using ^6^ ^0^Co irradiation before use. To avoid interference from the 3D structure of the porous T‐shaped scaffold in fluorescence detection, immunofluorescence (IF) staining experiments were conducted with cells cultured in the lower chamber and scaffold in the upper transwell chamber. For all other experiments, the cells were directly seeded onto scaffold surfaces. During culture, fresh medium was replaced every 48 h. For the TPBE+NIR group, the first NIR irradiation (808 nm, 1.0 W/cm^2^, 10 min) was first performed 3 h after seeding, followed by daily repetitions to maintain the scaffold surface temperature at 40–42°C [27].

#### 2.4.2 Cell Biocompatibility Assessment of Scaffolds

rBMSCs, HUVECs, and PC12 cells were used for all experiments. For proliferation assays, the cells were seeded onto scaffolds at 1 × 10^5^ cells per well in 24‐well plates. After 1, 4, and 7 days, 10% of the cell counting kit‐8 (CCK‐8) solution (C0038, Beyotime, China) was added to each well and incubated for 1 h. Absorbance at 450 nm was measured using a microplate reader (Thermo 3001, Thermo Scientific, USA), and growth curves were plotted. For live/dead staining, the cells were seeded at 3 × 10^4^ cells per well in 12‐well plates with scaffolds placed in the upper chamber. After three days, the cells were stained using the calcein‐AM/PI dual‐staining kit (Solarbio, China) and imaged using fluorescence microscope (CKX‐41, Olympus, Japan); the survival rates were quantified using the ImageJ software. To assess the effect of scaffold leachates on cell adhesion, the cells were seeded at 3 × 10^4^ cells per well in 12‐well plates. After 24 h, the medium was removed, and the cells were washed thrice using 1×PBS, fixed with 4% paraformaldehyde (Solarbio, China), and incubated with anti‐Vinculin primary antibody (ab129002, 1:200, Abcam). The cells were then incubated for 1 h at room temperature in the dark fluorescent secondary antibodies (Alexa Fluor488, Ab150077; Alexa Fluor594, Ab150080, 1:500, Abcam), and subsequently stained using fluorescein isothiocyanate (FITC)‐phalloidin (YF488‐Phalloidin, YP0059L; YF594‐Phalloidin, YP0052L, 1:200, Beyotime, China) and 4′,6‐diamidino‐2‐phenylindole dihydrochloride (DAPI; C1006, Beyotime, China) to label the cytoskeleton and cell nuclei, respectively. To evaluate the effects of different scaffold materials on intracellular reactive oxygen species (ROS) levels, experimental cells were inoculated into culture plates containing T, TPBE, and TPBE+NIR scaffolds. After 72 h of culture, the samples were washed with PBS. Intracellular ROS levels were detected using an active oxygen detection kit (S0033S, Beyotime), whereas nuclear staining was performed using Hoechst 33342 (C1025, Beyotime). The positive control group underwent pretreatment with 0.5 mm H_2_O_2_ for 20 min followed by identical staining. Fluorescent images were captured using a fluorescence microscope and quantified using the ImageJ software.

#### 2.4.3 Neurogenesis Capacity Evaluation of Scaffolds

For scratch wound assays, cells were seeded at 3 × 10^4^ cells per well in 12‐well plates (lower transwell chamber), with scaffolds placed in the upper chamber. Upon reaching confluence, a straight scratch was drawn using a P200 pipette tip. After washing the cells with PBS, a serum‐free medium was added for continued culture. Scratch areas were observed at 0 and 12 h using live‐cell staining. The healing rate was quantified using ImageJ.

Reverse transcription quantitative polymerase chain reaction (RT‐qPCR) was employed to evaluate neural differentiation‐related gene expression. PC12 cells were seeded onto scaffolds at 2 × 10^5^ cells per well and cocultured for 72 h. Total RNA was extracted using a trizol RNA extraction kit (Invitrogen, Carlsbad, CA) and reverse‐transcribed into cDNA using a PrimeScript RT Master Mix reverse transcription kit (Takara Bio Inc., Otsu, Shiga, Japan) following the instructions of the manufacturer. Glyceraldehyde‐3‐phosphate dehydrogenase (GAPDH; A19056, 1:150 000, ABclonal) served as the reference gene. Relative mRNA levels of neuron‐specific class III beta‐tubulin (TUj1), microtubule‐associated protein 2 (MAP2), calcitonin gene‐related polypeptide (CGRP), glial fibrillary acidic protein (GFAP), and nestin were calculated using the 2^(–ΔΔCt) method. All experiments were performed in triplicate (primers are presented in Table S1).

Western blotting was conducted to assess MAP2 protein expression in PC12 cells from different groups. These cells were lysed using a radioimmunoprecipitation assay buffer, whereas the proteins were separated using sodium dodecyl sulfate polyacrylamide gel electrophoresis, transferred to polyvinylidene fluoride membranes, and incubated overnight at 4°C using primary antibodies (MAP2, 17490‐1‐AP, 1:20 000, Proteintech; β‐actin, AC026, 1:500 000, ABclonal). After washing, the membranes were incubated with the corresponding secondary antibodies (1:1000) for 1 h. Signals were developed using enhanced chemiluminescence, imaged using a ChemiDoc CRS system (Bio‐Rad), and quantified using ImageJ.

IF staining was performed to assess the expression of neural differentiation proteins (CGRP and MAP2) in PC12 cells across different groups. First, the cells were seeded at 3 × 10^4^ cells per well in 12‐well plates, with scaffolds in the upper transwell chamber. After 72 h, the cells were fixed with 4% paraformaldehyde (15 min), permeabilized with 0.1% triton X‐100 (10 min), and blocked with 3% bovine serum albumin (1 h). The samples were incubated overnight at 4°C with primary antibodies (CGRP, ab81887, 1:100, Abcam; MAP2, 17490‐1‐AP, 1:500, Proteintech). Using the aforementioned identical culture method, DRG neurons were seeded at a density of 3 × 10^4^ cells per well in a 12‐well plate coated with a poly‐l‐lysine solution (P2100, Solarbio). After seven days of culture, the samples were fixed with 4% polyformaldehyde and subsequently immunofluorescently stained with Tuj1 antibody (MA1‐118, 1:400, Invitrogen) and NeuN antibody (YP‐rAb‐17486, 1:500, Proteintech). The following day, after being washed using PBS, the samples were incubated with species‐specific secondary antibodies in the dark at room temperature for 1 h. The cell nuclei were counterstained using DAPI, the images were captured under a fluorescence microscope, and the analysis was performed using ImageJ.

#### 2.4.4 Mechanistic Analysis of Scaffolds Promoting Neurogenic Activity of PC12 Cells

To investigate how TPBE+NIR scaffolds enhance PC12 cell neurodifferentiation, mRNA transcriptome sequencing was performed on cell (*n* = 3) directly and cocultured with T and TPBE+NIR scaffolds for seven days. Total RNA was extracted using the trizol kit, and after quality verification, library construction was outsourced to Wuhan Sevier Biotechnology. Dual‐end sequencing was conducted on an Illumina NovaSeq 6000 platform. Differentially expressed genes (DEGs) were identified using DESeq2 with |log_2_ (fold change) |> 1 and adjusted *p* < 0.05. Gene ontology (GO) functional annotation and Kyoto Encyclopedia of Genes and Genomes (KEGG) pathway enrichment analyses were performed for DEGs (significant enrichment: adjusted *p* < 0.05).

To clarify whether TPBE+NIR‐mediated neuronal differentiation and neurite formation depend on the PI3K/Akt signaling pathway, intervention with the PI3K inhibitor LY294002 (T2008, TargetMol) was employed. The experimental groups were as follows: T, TPBE+NIR, and TPBE+NIR/LY294002. After cell seeding, the TPBE+NIR/LY294002 group was supplemented with the LY294002 (20 µm) inhibitor. Detection of PI3K (41339, 1:1000, SAB), phospho‐PI3K (12057, 1:1000, SAB), AKT (121155, 1:1000, SAB), phospho‐AKT (11124, 1:1000, SAB), and MAP2‐related protein expression were performed as described in Section 2.4.3.

#### 2.4.5 Angiogenesis Capacity Evaluation of Scaffolds

In cell migration assay, the cells were seeded at 2 × 10^4^ cells per well in the transwell upper chamber (PTEP12H48, Corning, USA). Scaffolds from each group were placed in the lower chamber, and serum‐free medium was added. After 12 h of incubation, the cells migrating through the transwell membrane were stained with 0.5% crystal violet (C0121, Beyotime, China) for 10 min and observed under a light microscope. Migrating cell numbers were quantified using ImageJ. Scratch wound assays were performed as described in Section 2.4.3.

In tube formation assay, the cells were cocultured directly with each scaffold group for 48 h. They were then harvested, resuspended as a single‐cell suspension, and seeded at 2 × 10^4^ cells per well into 24‐well plates precoated with Matrigel (356230, Corning, USA), followed by a 45‐min incubation. After 6 h, the formed tubular structures were stained with calcein‐AM and imaged using fluorescence microscopy. Quantitative analysis was performed using ImageJ.

In gene and protein expression analyses, the cells were cocultured directly with scaffolds at 2 × 10^5^ cells per well for 72 h. The mRNA levels of hypoxia‐inducible factor‐1α (HIF‐1α), platelet‐derived growth factor receptor (CD31), and vascular endothelial growth factor (VEGFA) were measured (primers are presented in Table S2). CD31 (80530‐1‐RR, 1:1000, Proteintech) and VEGFA (GB11034B, 1:1000, Servicebio) were analyzed through Western blotting, using GAPDH (GB15004, 1:10000, Servicebio) as the housekeeping control. IF staining was performed to visualize VEGFA (66828‐1‐Ig, 1:400, Proteintech) and CD31 (80530‐1‐RR, 1:800, Proteintech) in scaffolds and cells after 72 h of coculture. Procedures followed those described in Section 2.4.3.

#### 2.4.6 Osteogenesis Capacity Evaluation of Scaffolds

To evaluate the regulatory effects of different scaffold materials on rBMSCs, osteogenic differentiation cells were cultured using direct (1 × 10^5^ cells per well) and indirect (3 × 10^4^ cells per well) contact methods in osteogenic induction medium (10% FBS, 1% P/S, 50 µg/mL ascorbic acid, 10 nm dexamethasone, and 10 mm sodium β‐glycerophosphate). After seven days, alkaline phosphatase (ALP) staining was performed using a 5‐bromo‐4‐chloro‐3‐indolyl‐phosphate/nitro blue tetrazolium ALP color development kit (C3206, Beyotime). ALP activity was quantified using an ALP activity assay kit (P0321, Beyotime) and a bicinchoninic acid protein assay kit (P0010, Beyotime) according to the manufacturer instructions. Mineral deposition was examined after 14 days using Alizarin red S staining (C0148S, Beyotime). To quantify calcium deposition, 10% cetylpyridinium chloride was added to each well to extract the mineralized matrix, whereas absorbance at 562 nm was measured using a microplate reader.

The cells were seeded onto the scaffold surface at 2 × 10^5^ cells per well. After 14 days of culture, mRNA expressions of bone morphogenetic protein 2 (BMP2), osteopontin (OPN), osteocalcin (OCN), and Type I collagen (COL1A1) were analyzed using RT‐qPCR (primers are presented in Table S3). Protein expressions of OCN (DF12303, 1:1000, Affinity) and COL1A1 (GB11022, 1:1000, Servicebio) were analyzed using Western blot, with β‐actin (GB15003, 1:5000, Servicebio) as the reference gene. IF staining was conducted to assess OCN (161517‐1‐AP, 1:200, Proteintech) and COL1A1 (SAB5700733, 1:200, Sigma) protein expressions in rBMSCs after 14 days of indirect coculture with scaffolds. Procedures were in accordance with the methods described in Section 2.4.3.

### 2.5 In Vivo Experiments

#### 2.5.1 Construction of Bilateral Femoral Condylar Bone Defects and Scaffold Implantation in Rabbits

We selected 45 healthy male New Zealand white rabbits, aged 6–8 weeks and weighing 2.5–3.5 kg. Animal procedures were approved by the Institutional Ethics Committee (Approval No.: XYLS2024120). Animals were randomly assigned to the T, TPBE, and TPBE+NIR groups. Surgery was performed under anesthesia induced via intravenous propofol (6–8 mg/kg) and maintained with 2.5% isoflurane inhalation. After the femoral condyle was exposed using a lateral patellar approach, a cylindrical bone defect (6 mm diameter and 10 mm depth) was created using a circular drill. The corresponding implant was inserted, and the wound was closed with layered sutures. Postoperatively, cefotaxime (10 mg/kg/day, subcutaneous injection) was administered for seven days to prevent infection. For the TPBE+NIR group, NIR intervention was performed every five days postoperatively. Local irradiation with an 808 nm laser (1.0 W/cm^2^) was performed for 10 min. Temperature was continuously monitored using an infrared thermal imaging camera and maintained at 40–42°C. The area was allowed to cool naturally after irradiation.

#### 2.5.2 Vascular Perfusion

At 2 and 4 weeks after implantation, two rabbits from each group were randomly selected for microvascular perfusion analysis according to the method described by Gao et al. [11]. Anesthetized rabbits underwent abdominal shaving and tissue dissection along the midline to fully expose the abdominal cavity. After careful dissection of the abdominal aorta and inferior vena cava, a 1.5 mm diameter infusion catheter was inserted and secured distally on the abdominal aorta, whereas the proximal vein was ligated to establish a closed perfusion circuit. Vessels were simultaneously flushed with sodium heparin (400 U/L) saline until effluent became clear, followed by perfusion with 10% paraformaldehyde for primary fixation. The MICROFIL (MV‐122) perfusion solution was then prepared according to the manufacturer instructions and infused into the lower limb vasculature (50 mL) using an automatic syringe pump at a constant flow rate of 2 mL/min. After perfusion, intact femoral specimens were harvested, fixed in 4% polyformaldehyde for 24 h, and then decalcified in 10% ethylenediaminetetraacetic acid for two months.

#### 2.5.3 Study on Neuro–Vascular–Bone Integration and Biosafety of Porous Titanium Scaffolds In Vivo

At weeks 2, 4, 8, and 12 after implantation, animals in each group were euthanized (*n* = 3/group/time point). Femoral samples were collected, fixed, and subjected to 3D bone regeneration evaluation within the scaffold, including vascular networks within the scaffold and surrounding 2 mm region of interest, using micro‐computed tomography (CT, SkyScan 1276, Bruker). Image reconstruction was performed using AYRecon software. Cylindrical regions of interest (ROIs; diameter = 6 mm and height = 10 mm) were established in VG Studio Max 3.5. Fixed thresholds (bone > 30500, vessels > 6500) were applied to generate 3D models of bone and vasculature. Quantitative analyses included bone volume fraction (BV/TV), blood vessel volume fraction (BVV/TV), trabecular bone thickness (Tb.Th), trabecular bone number (Tb.N), trabecular bone separation (Tb.Sp), and vessel diameter.

Samples after micro‐CT scanning were subjected to graded dehydration and resin embedding. Initial 150 µm sections were prepared using a hard‐tissue sectioning system (EXAKT 312, EXAKT company, Germany) and then polished to 50 µm using a precision polishing system (EXAKT 400CS, EXAKT company, Germany). Vascular perfusion samples were imaged using a fluorescence microscope (Leica M205 FA, Leica, Germany) for green and red fluorescent protein visualization, and vessel diameter and density were quantified using ImageJ. For nonperfused samples, sections from weeks 2 and 4 were subjected to IF staining to assess neural (CGRP) and vascular (CD31) marker expressions. Sections from weeks 4, 8, and 12 underwent Van Gieson (VG) and Masson trichrome staining (Senbega, China). Sections were scanned using a digital pathology slide scanner (KF‐FL‐020, KFBIO, Jiangfeng Bio) for histology observation. Bone volume fraction (BV/TV) was calculated as the ratio of bone tissue volume to total scaffold volume. Bone‐implant contact (BIC), denoted as the percentage of implant surface contacting bone without intervening soft tissue layers, was quantified as the ratio of the bone‐contact length to the total scaffold surface length. Additionally, osteogenic markers (OCN and COL1A1) were assessed using IF staining according to standard protocols. All images were quantitatively analyzed using the ImageJ software. To assess systemic biocompatibility, blood samples were collected at week 12 after implantation for routine hematology testing. Major organs, including the heart, liver, spleen, lungs, and kidneys, were harvested for hematoxylin‐eosin staining to analyze pathological and inflammatory responses.

#### 2.5.4 Statistical Analysis

All data in the study were presented as mean ± standard. Comparisons among three or more groups were conducted using one‐way analysis of variance, whereas comparisons between two groups were analyzed using Student's *t*‐test. Statistical significance was defined as *p* < 0.05 and indicated as *p < 0.05. All data in this study were analyzed using the GraphPad Prism v.8 and Origin Pro software (v9.0, Origin Labs, USA).

## 3 Results and Discussion

### 3.1 Preparation and Characterization of BPNSs and PDA@(BP+Eu) Nanosheets

**FIGURE 2:**
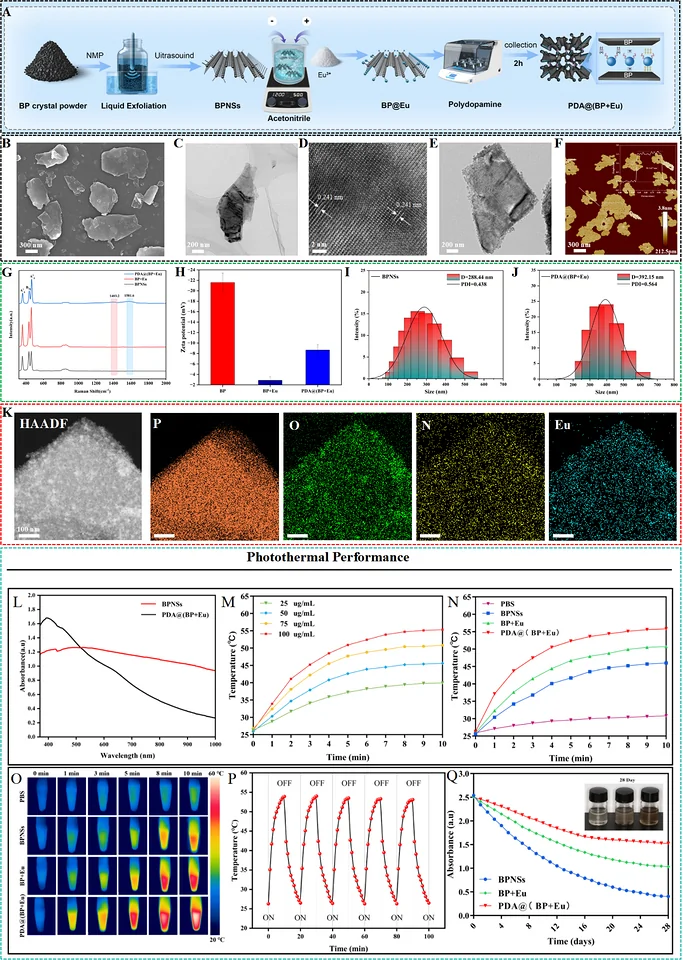
Characterization of BPNSs and BP+Eu and PDA@(BP+Eu) nanosheets. (A) Schematic of PDA@(BP+Eu) fabrication; (B) SEM images of BPNSs (scale bar = 300 nm); (C,D) TEM and High‐resolution images of BPNSs (scale bar = 200 and 2 nm, respectively); (E) TEM images of PDA@(BP+Eu) (scale bar = 200 nm); (F) AFM image and the corresponding height profile of BPNSs (scale bar = 300 nm); (G) Raman spectra of BPNSs, BP+Eu, and PDA@(BP+Eu); (H) Zeta potential of BPNSs, BP+Eu, and PDA@(BP+Eu); (I) Average size of BPNSs; (J) PDA@(BP+Eu) nanosheets; (K) TEM image and elemental mapping of PDA@(BP+Eu) nanosheets (scale bar = 100 nm); (L) UV–vis absorption spectra of BPNSs and PDA@(BP+Eu) nanosheets in aqueous dispersions; (M) Photothermal‐heating curves of BPNSs at different concentrations under 808 nm laser irradiation (1.0 W/cm^2^); (N) Heating curves of samples in vitro and; (O) Infrared thermal images of PBS, BPNSs, BP+Eu nanosheets, and PDA@(BP+Eu) nanosheets in aqueous dispersion under NIR irradiation; (P) On/OFF photothermal heating curves of PDA@(BP+Eu) nanosheets under five cycles of laser irradiation; (Q) UV–vis absorption spectra of BPNSs, BP+Eu, and PDA@(BP+Eu) dispersed in PBS exposed to air for 0, 4, 8, 12, 16, 20, 24, and 28 days. Data are expressed as mean ± standard deviation (SD) (*n* ≥ 3).

In bone tissue engineering, the development of nanomaterials with controllable structure and stable properties remains crucial for enhancing bone regeneration. SEM and TEM images show that the synthesized BPNSs exhibited uniform sheet‐like morphology with well‐defined edges and lateral dimensions of approximately 200 nm (Figure 2B,C). After element mapping of selected BPNSs (Figure S1), P was found to be evenly distributed throughout the entire nanosheets, thus confirming the successful preparation of BPNSs. High‐resolution TEM further confirmed structural integrity by revealing lattice fringes of 0.241 nm, corresponding to the interplanar spacing of orthorhombic BP crystals (Figure 2D) [28]. AFM analysis indicated a nanosheet thickness of approximately 1.67 nm, corresponding to a 2D nanomaterial composed of thin layers of phosphorene (Figure 2F) [29]. These few‐layer BPNSs offer an excellent interface for cell adhesion and bioactive molecule loading owing to their larger specific surface area, increased edge‐active sites, and controllable degradation behavior [30, 31].

To simultaneously enhance the BPNSs stability, a PDA‐coated composite nanostructure with P–Eu coordination (PDA@(BP+Eu)) was designed and synthesized (Figure 2A). To optimize the BP–Eu nanosheet synthesis, the Eu/BPNSs feed ratios were varied, whereas the Eu:P elemental ratio was quantified using ICP‐OES to determine the maximum Eu^3+^ loading capacity. As shown in Figure S2, Eu loading increases with higher feed ratios and reaches saturation at a ratio of 8:1. Consequently, this ratio was utilized for subsequent preparation. Zeta potential analysis indicated that Eu^3^^+^ modification significantly increased the surface potential of BPNSs from –21.58 ± 1.81 to –2.86 ± 0.71 mV, indicating charge neutralization through coordination interactions [32]. After PDA coating, the zeta potential of the PDA@(BP+Eu) nanocomposite system was further adjusted to –8.66 ± 1.02 mV (Figure 2H). The particle size of BPNSs sharply increased from 288.44 ± 2.91 to 392.15 ± 2.22 nm (Figure 2I,J), confirming successful construction of PDA@(BP+Eu) nanosheets. The polydispersity index (PDI), which indicates nanomaterial uniformity, was 0.438 ± 0.04 for BPNSs and 0.564 ± 0.02 for PDA@(BP+Eu) nanosheets (Figure 2I,J). The increased PDI of PDA@(BP+Eu) was attributed to the tendency of PDA to form nanospheres during polymerization, resulting in partial aggregation and a slight decrease in dispersion uniformity [33]. TEM revealed uniformly distributed dot‐like composites on the PDA@(BP+Eu) nanosheet surface (Figure 2E), whereas elemental mapping confirmed the presence of P, O, N, and Eu (Figure 2K), indicating successful Eu and PDA incorporation onto the BPNSs. Raman spectroscopy (Figure 2G) exhibited characteristic peaks of pristine BPNSs at 360.3, 433.6, and 461.3 cm^−^^1^, corresponding to A^1^g (out‐of‐plane), B_2_g, and A^2^g (in‐plane) vibrational modes, respectively [34]. After Eu^3^^+^ modification, these peaks exhibited redshifts, indicating coordination bonding between Eu^3^^+^ and BPNSs, which may alter BP electron cloud distribution [16]. The PDA@(BP+Eu) nanosheets exhibited characteristic PDA peaks at 1403.2 and 1581.6 cm^−^^1^, corresponding to the breathing vibration of the PDA aromatic ring and stretching vibration of the C═C conjugated backbone, respectively [35], confirming successful encapsulation and preservation of the π–π conjugated structure. Further, blueshifts in the A^1^g and A^2^g peaks of BPNSs indicate reduced layer number and weakened interlayer coupling, respectively, suggesting an ultrathin structure [36, 37]. Throughout the process, the A^1^g/A^2^g peak intensity ratio remained stable at approximately 0.78 (above the oxidation threshold of 0.6) [38], confirming BPNSs crystal structure integrity without considerable oxidation, thereby facilitating their stable application in physiological environments. These results confirm the successful synthesis of PDA@(BP+Eu) nanosheets.

Mild photothermal therapy has recently garnered considerable attention in bone repair owing to its noninvasive nature, remote controllability, and excellent tissue penetration [39]. Hence, the photothermal performance of BPNSs and their composite systems were evaluated. UV–vis spectroscopy revealed a prominent absorption peak at 528 nm for BPNSs dispersion, whereas PDA@(BP+Eu) nanosheets exhibited enhanced absorption at approximately 395 nm, indicating improved visible‐light‐harvesting after PDA modification (Figure 2L) [40]. Under NIR irradiation, BPNSs exhibited concentration‐dependent heating, whereas PDA@(BP+Eu) nanosheets achieved optimal photothermal performance (Figure 2M–O). Stable temperature elevation was maintained over five irradiation cycles without considerable performance degradation (Figure 2P). These results confirm the photothermal activity of BPNSs and indicate that Eu^3^^+^ modification and PDA coating enhance photothermal conversion efficiency and cycling stability.

To verify practical reliability, the stabilities of BPNSs, BP+Eu, and PDA@(BP+Eu) dispersions were monitored at room temperature for 28 days. As shown in Figures 2Q and S3, the BPNS dispersion is nearly transparent on day 28, with absorbance decreasing considerably over time, indicating susceptibility to oxidation in aqueous solutions. By contrast, the Eu^3^^+^‐modified dispersion exhibits a slower decrease in absorbance, owing to Eu^3^^+^ passivating surface active sites via cation–π interactions, thereby retarding oxidation [19]. The PDA@(BP+Eu) nanosheets exhibit the most gradual absorbance change, with no noticeable alteration in dispersion appearance, confirming that the PDA coating effectively shields the nanosheets from water and oxygen [24], thereby enhancing long‐term stability. Overall, the synergistic combination of Eu^3^^+^ coordination passivation and PDA surface coating produces PDA@(BP+Eu) nanosheets with high photothermal efficiency and excellent chemical stability.

### 3.2 Preparation and Characterization of TPBE Scaffold

**FIGURE 3:**
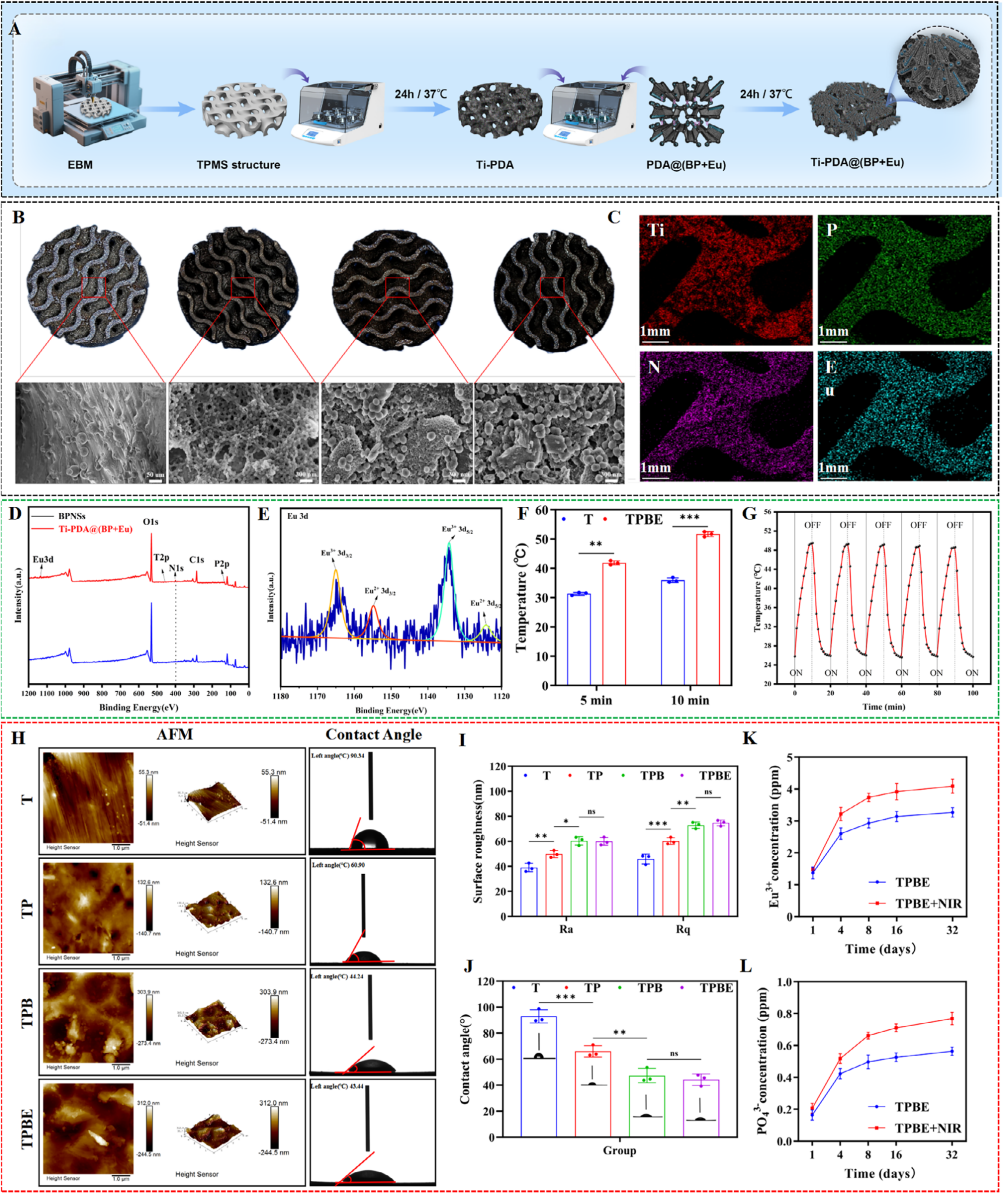
Characterizations of samples: (A) Schematic of TPBE fabrication. (B) Digital and SEM images of T, TP, TPB, and TPBE (scale bar = 50 µm and 300 nm). (C) EDS spectra and elemental mapping of TPBE (scale bar = 1 mm). (D) XPS spectra of BPNSs and TPBE. (E) High‐resolution Eu spectra of the TPBE surface coating. (F) Maximum surface temperatures of T and TPBE during 5–10 min irradiation. (G) Photothermal stability test. (H) Surface roughness and water contact angles of the scaffolds. (I,J) Statistical analyses of surface roughness and water contact angles across different groups. (K,L) Cumulative Eu^3+^ and PO_4_^3−^ release concentrations from the TPBE and TPBE+NIR groups, respectively. Data are expressed as mean ± SD (*n* ≥ 3). ns: not significant, *p < 0.05, **p < 0.01, ***p < 0.001.

A biomimetic scaffold incorporating PDA@(BP+Eu) nanosheets was prepared using a two‐step strategy (Figure 3A). As it was a load‐bearing implant, its mechanical reliability was crucial. Compression testing (Figure S4) indicated that the TPMS‐structured scaffold exhibited a compressive strength of 396.23 MPa, considerably higher than that of cancellous bone (2–20 MPa) [41], thereby ensuring effective mechanical support at the implantation site [42]. The elastic modulus was 5.24 GPa, which was similar to the cortical bone modulus range (2–20 GPa) [43], thereby enabling stiffness matching between the implant and bone tissue and reducing stress shielding effects [44]. Hence, the TPMS scaffold demonstrated excellent mechanical compatibility, characterized by high compressive strength and structural stability. SEM images revealed that BPNSs and PDA nanoparticles were interwoven and uniformly encapsulated on the TPBE scaffold surface, forming a continuous nanoscale coating (Figure 3B). EDS mapping confirmed uniform distributions of Ti, P, N, and Eu across the surface (Figure 3C), whereas the colocalization of P, N, and Eu signals verified the successful incorporation of PDA@(BP+Eu) nanocomposites. XPS analysis confirmed the chemical composition of the TPBE scaffold. Characteristic P, Eu, and N peaks were observed in the survey spectrum (Figure 3D). The 3d high‐resolution spectrum of Eu exhibited peaks at 1165.0/1154.8 and 1134.3/1124.2 eV, corresponding to the 3d_5_/_2_ and 3d_3_/_2_ orbitals of Eu(III) and Eu(II), respectively (Figure 3E), which indicated partial reduction of Eu^3^^+^ to Eu^2^^+^ by the reductive properties of BPNSs [45]. This valence transition enhanced Eu–P coordination and improved BPNSs stability under physiological conditions. The results confirmed the formation of stable Eu–BP chemical interactions and the robust immobilization of PDA@(BP+Eu) nanosheets on the titanium substrate via the PDA interfacial layer.

The TPBE scaffold exhibited excellent surface physicochemical properties and photothermal response characteristics. The AFM analysis indicated that BPNSs and PDA incorporation considerably increased surface roughness (Figure 3H,I). Similarly, owing to the modification of the stent surface coating, the water contact angles on the surfaces of all sample groups gradually decreased, with the TPB and TPBE groups exhibiting the lowest values (Figure 3H,J), indicating hydrophilicity. The improvements were attributed to the abundant polar groups in PDA and weak interlayer van der Waals forces of BPNSs, which facilitated oxidation of edge phosphorus atoms to form hydrophilic groups, such as P─OH and P═O [46, 47]. After 10 min of NIR irradiation, the peak surface temperatures of T and TPBE reached 35.97 ± 0.81 and 51.67 ± 0.85°C, respectively. Notably, TPBE achieved an average temperature of 41.90 ± 0.65°C within 5 min of irradiation, demonstrating rapid and efficient photothermal conversion (Figure 3F). Further intermittent irradiation cycling tests (10‐min irradiation/10‐min cooling for five cycles) showed that the TPBE scaffold temperature remained stable at 49.1 ± 0.35°C without considerable decay (Figure 3G). This confirmed that the introduction of PDA@(BP+Eu) nanosheets onto the T scaffold surface conferred both superior photothermal responsiveness and excellent photothermal stability. To further validate the stability of the PDA@(BP+Eu) nanocoating on the TPBE scaffold surface, the samples were subjected to sterilization and five photothermal cycles, followed by multidimensional characterization using SEM, EDS, Raman spectroscopy, and photothermal performance assays. The SEM results showed that the coating remained intact, continuous, and uniformly distributed across all treated groups without cracks, delamination, or aggregation (Figure S5A). The EDS elemental analysis revealed uniform distribution of characteristic elements P, Eu, and N across all three scaffold groups (Figure S5A). The Raman spectroscopy showed consistent positions, shapes, and relative intensities for the A^1^g, B_2_g, and A^2^g peaks of BPNSs, indicating no significant oxidative degradation (Figure S5C). The photothermal performance tests indicated that although the maximum temperature increase under 10 min of NIR irradiation decreased slightly after treatment compared with those of initial samples, no statistically significant differences were observed between groups (*p* > 0.05) (Figure S5B). These results collectively confirmed that the PDA@(BP+Eu) coating remained stable after sterilization and multiple NIR treatments, with no notable delamination, excessive BPNSs degradation, or significant europium loss; thus, this coating's resistance to damage during conventional sterilization and repeated NIR exposure was verified. The results of the ICP‐OES analysis (Figure 3K,L) demonstrated that the Eu^3+^ release from the TPBE scaffold exhibited a characteristic two‐phase pattern: the first eight days represented a rapid release phase with a cumulative release of 30.09 ± 1.49 µg, followed by a plateau phase; a total cumulative release of 34.71 ± 1.51 µg during the days 1–32. By contrast, the TPBE+NIR group exhibited higher Eu^3+^ release levels at all time points compared with the TPBE group, with a cumulative release of 38.32 ± 1.32 µg during the first eight days, followed by a plateau phase; a total cumulative release of 43.37 ± 2.30 µg during the days 1–32. The PO_4_^3−^ released from the oxidative degradation of BPNSs within the TPBE+NIR scaffold followed a release trend similar to that of Eu^3+^: a cumulative release of 6.80 ± 0.23 µg during the first eight days, followed by a plateau phase; a total cumulative release of 8.11 ± 0.38 µg during the days 1–32. This behavior was attributed to the photothermal effect between PDA and BP under NIR irradiation, which induces localized heating that promotes the oxidative degradation of BPNSs and subsequent release of PO_4_^3−^, accompanied by dissociation of the surface‐loaded Eu^3+^ [19, 24, 48].

### 3.3 In Vitro Cytocompatibility Evaluation of Scaffolds in Each Group

**FIGURE 4:**
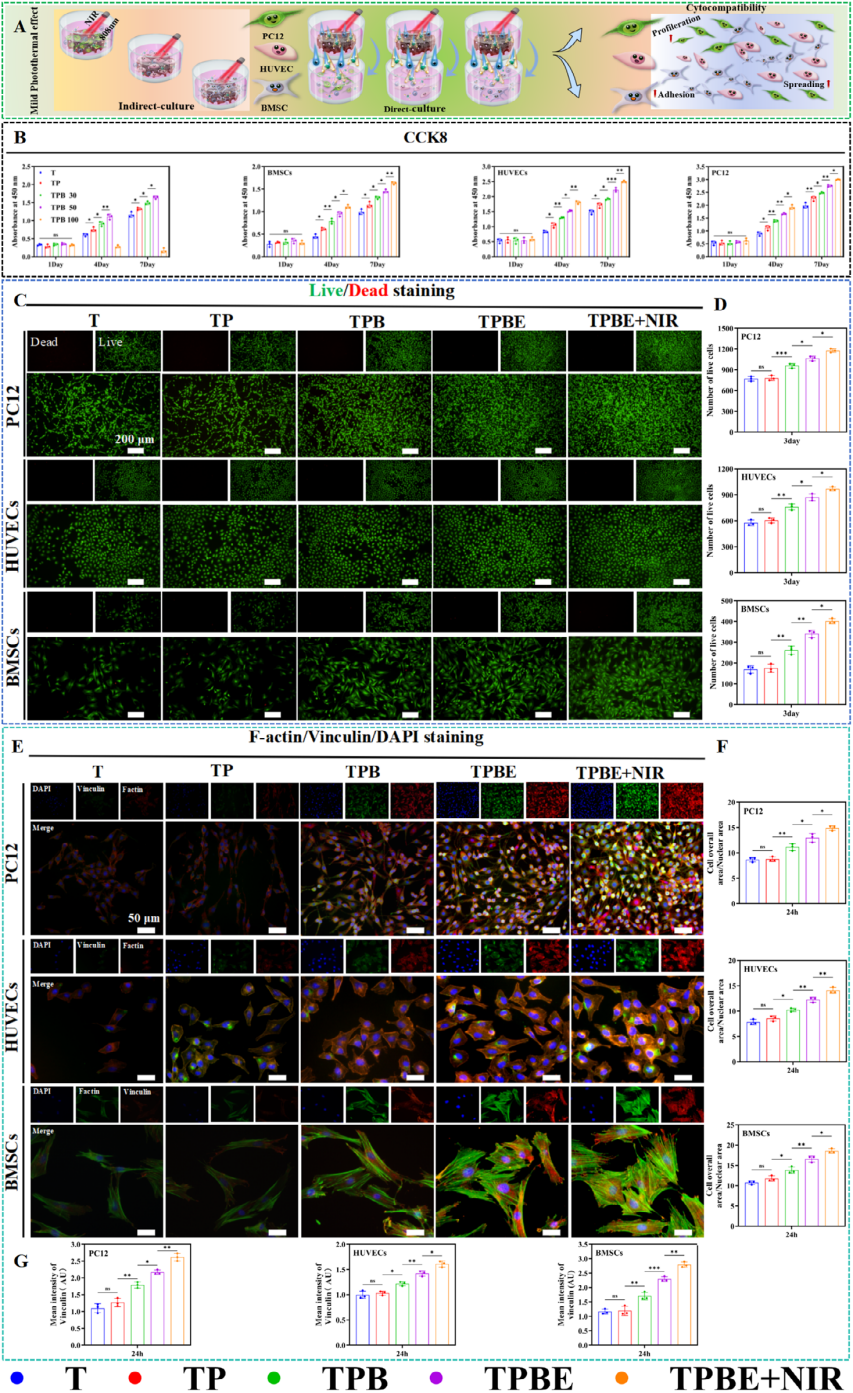
In vitro biocompatibility of scaffolds in different groups: (A) Schematic of the cell‐scaffold coculture system under NIR irradiation. (B) Cell viability of rBMSCs, HUVECs, and PC12 cells cultured on different scaffolds for 1, 4, and 7 days, assessed using the CCK‐8 assay kit. (C) Live/Dead staining of after 72 h of indirect coculture (scale bar = 200 µm), live cells (green: calcein‐AM) and dead cells (red: PI) visualized using fluorescence microscopy. (D) Quantification of live/dead staining. (E) IF staining of F‐actin and vinculin after 24 h (scale bar = 50 µm). (F,G) Quantitative analysis of the cell‐to‐nuclear area ratio (CN ratio) and Vinculin fluorescence intensity. Data are expressed as mean ± SD (*n* ≥ 3). ns: not significant, *p < 0.05, **p < 0.01, ***p < 0.001.

In vitro cell compatibility of the scaffold is crucial for subsequent in vivo animal experiments. In this study, HUVECs, rBMSCs, and PC12 cells were used to evaluate the cell viability and proliferation capacity of T‐scaffolds before and after modification, with and without NIR irradiation (Figures 4A and S6). Although PDA coating exhibits excellent biocompatibility [49], the biological effects of BPNSs, which are emerging 2D materials, are concentration dependent [50, 51]. Hence, the safe loading concentration was screened using a CCK‐8 assay kit. As shown in Figure 4B, rBMSCs exhibit optimal proliferation activity on days 4 and 7 when loaded with 50 µg/mL (TPB50 group). This indicates that the incorporation of BPNSs creates a superior extracellular matrix (ECM)‐mimetic microenvironment that expands cellular growth space and promotes cell adhesion and proliferation [52]. Conversely, 100 µg/mL (TPB100 group) inhibits cell growth. Hence, 50 µg/mL was selected as the safe loading concentration and used for subsequent Eu^3^^+^ functionalization.

Although Eu^3^^+^ is widely applied as an osteogenic factor in bone repair, its cellular safety requires further validation [21]. Based on the established safe BPNSs concentration, the biocompatibility of Eu^3^^+^‐modified TPBE scaffolds was evaluated using three key cell types (rBMSCs, HUVECs, and PC12 cells). The CCK‐8 assay results (Figure 4B) showed that TPBE scaffolds considerably enhanced proliferation of all three cell types than the control scaffolds. This effect was attributed to the time‐dependent release of Eu^3^^+^/PO_4_^3−^, which generated a microenvironment conducive to cellular metabolism and growth [53]. Furthermore, proliferation was further enhanced under NIR irradiation, indicating that TPBE scaffolds combined with NIR‐assisted mild hyperthermia synergistically promote cell growth. This finding was consistent with those of previous studies, which demonstrated that mild photothermal effects facilitate proliferation, tissue regeneration, and osteogenic activity [54]. Further, live/dead staining confirmed these results. The TPBE+NIR group exhibited the highest number of live cells followed by the TPBE group (Figure 4C,D), indicating that the PDA@(BP+Eu) nanosheet core unit on the scaffold surface provided additional bioactive cues that supported proliferation, whereas the NIR‐derived mild photothermal therapy further amplified this benefit. Owing to the inherent susceptibility of BPNSs to oxidation and the potential risk of NIR‐mediated photothermal stimulation that induces cellular oxidative stress, this study used a 2′,7′‐dichlorodihydrofluorescein diacetate (DCFH‐DA) fluorescent probe to measure intracellular ROS levels in PC12, HUVEC, and rBMSCs after various treatments. This evaluated the oxidative stress safety of the TPBE scaffold combined with the NIR treatment. As shown in Figure S7A,B, the TPBE and TPBE+NIR groups exhibit slight increases in the intracellular green fluorescence signals compared with the T group; however, these differences are not statistically significant (*p* > 0.05). By contrast, when H_2_O_2_ is used as a positive damage control, the intracellular fluorescence signals in both the TPBE and TPBE+NIR groups significantly decrease (*p* < 0.0001). Moreover, existing research has confirmed this that BPNSs possess efficient ROS scavenging properties and good biological activity [40]. PDA numerous catechol groups, effectively eliminates intracellular ROS, whereas the mild photothermal effect of NIR irradiation (41 ± 1°C) further enhances their ROS scavenging efficiency [40, 55]. Therefore, the likelihood of sublethal oxidative damage induced by BPNSs degradation or photothermal stimulation is low, thereby further reinforcing the safety of the TPBE+NIR treatment regimen.

To investigate cell adhesion behavior, immunofluorescent staining for F‐actin, focal adhesion proteins, and nuclei was performed on all three cell types. Compared with the T and TP groups, the TPB group exhibited more extensive cell spreading across all three cell types, indicating that PO_4_^3^^−^ released from BPNSs promotes cytoskeletal reorganization and expansion [56]. Eu^3^^+^ modification (TPBE group) further improved cell spreading, whereas the TPBE+NIR group demonstrated synergistically enhanced spreading morphology (Figure 4E). Consequently, quantitative analysis of the cell nuclear area ratio demonstrated considerably higher values in the TPBE+NIR group than the other groups (*p* < 0.05) (Figure 4F), confirming the synergistic contribution of cell spreading by photothermal stimulation. Furthermore, vinculin expression was considerably upregulated in the TPBE+NIR group, exhibiting a characteristic “cytoplasmic diffusion‐edge enrichment” pattern (Figure 4G), indicative of focal adhesion maturation and enhanced cell‐material interactions [57]. These results demonstrate that the TPBE scaffold, particularly under NIR treatment, exhibits excellent in vitro biocompatibility and strong potential for bone tissue regeneration.

### 3.4 Effects of Scaffolds in Each Group on Neurogenesis of In Vitro PC12 Cells

**FIGURE 5:**
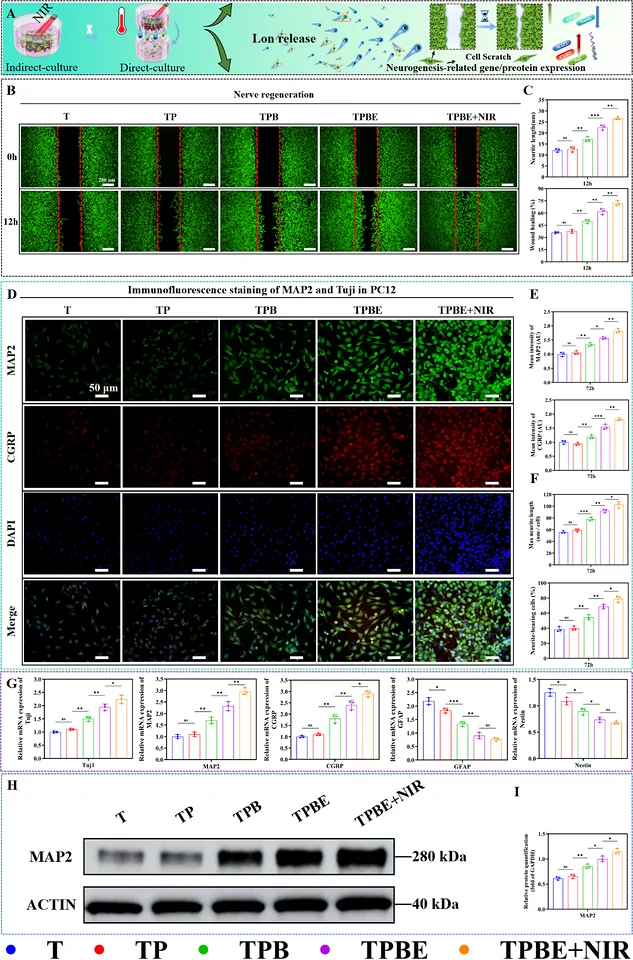
Effects of NIR irradiation on neurite outgrowth and neural differentiation in PC12 cells cultured on different scaffolds: (A) Schematic of the PC12‐scaffold coculture system under NIR irradiation and subsequent induction of neuroregeneration. (B) Representative images of the scratch wound assay; (C) Neurite length and migration rate (%) (scale bar = 200 µm). (D,E) IF staining and quantitative analysis of CGRP and MAP2 after 72 h of indirect coculture (scale bar = 50 µm). (F) Maximum neurite length per cell and percentage of neurite‐bearing cells. (G) RT‐qPCR analysis of neuroregeneration‐related genes (TUj1, MAP2, Nestin, GFAP, and CGRP) after 72 h of direct contact. (H,I) Western blot analysis of neuroregeneration‐related proteins (MAP2) and quantitative results after 72 h of direct contact. Data are expressed as mean ± SD (*n* ≥ 3). ns: not significant, *p < 0.05, **p < 0.01, ***p < 0.001.

Bone defect repair involves numerous complex biological processes, including innervation, angiogenesis, and osteoblastogenesis, with innervation normally occurring first and sequentially regulating subsequent regeneration processes [58]. Given the critical role of peripheral nerve fibers in the repair process of bone defect areas, PC12 cells from each group were indirectly coculture using scaffolds to analyze the influence of different microenvironments on axonal growth (Figure 5A). Scratch wound assays demonstrated that, compared with the T and TP groups, the TPB scaffold considerably enhanced PC12 cell migration and axonal growth (Figure 5B,C). This effect was primarily attributed to the conductive interface provided by BPNSs, which mimicked the native neurophysiological microenvironment [59]. Additionally, the PO_4_^3^^−^ released during its degradation provided raw materials for matrix mineralization and facilitated nerve regeneration [60]. Notably, the TPBE+NIR scaffold exhibited the most pronounced migration‐promoting effect, followed by the TPBE scaffold. This indicates that NIR‐triggered controlled Eu^3^^+^ release, combined with mild photothermal stimulation, synergistically enhances PC12 neurodifferentiation [61, 62]. This can further promote direct cell migration, providing favorable conditions for guiding nerve fiber extension into bone defect areas.

To elucidate the underlying molecular regulatory mechanism, the expression of neurodifferentiation‐related markers was analyzed. The RT‐qPCR results revealed that the TPBE scaffold considerably upregulated neuronal markers Tuj1, MAP2, and CGRP, while considerably downregulating the neural stem/progenitor cell marker Nestin and astrocyte marker GFAP. These effects were further enhanced under mild NIR irradiation (Figure 5G). The Western blot analysis confirmed that MAP2 protein expression was highest in the TPBE+NIR group, followed by the TPBE group (Figure 5H,I). IF staining revealed stronger CGRP and MAP2 signals in the TPBE+NIR groups (Figure 5D,E). Furthermore, the quantitative analysis of neuronal morphology demonstrated that the TPBE+NIR group exhibited significantly superior values in PC12 cells compared to other groups in terms of maximum axon length, percentage of axons carrying cells (Figure 5F), total axonal length per cell, number of branching points, and average axon length (Figure S8A–C). As axonal regeneration is a critical step in peripheral nerve regeneration [63], these findings preliminarily suggest that the combination of TPBE scaffolds with NIR photothermal stimulation effectively promotes axonal growth. However, PC12 cell lines lack sensory neuron‐specific subtype markers and certain synaptic plasticity characteristics [64]. To address this, we used IF staining on primary DRG neurons from mice. The results showed that the TPBE group exhibited stronger Tuj1 (green) and NeuN (red) fluorescence signals compared to the T and TP groups, followed by the TPB group. This effect was further enhanced after NIR irradiation (Figure S9A). Results of the quantitative analysis confirmed that the proportion of NeuN‐positive cells and fluorescence intensity levels in the DRG cells of the TPBE+NIR group were significantly higher than those in other groups (Figure S9C). Additional data on maximum axon length, percentage of axons carrying cells, total length, number of branching points, and average length further supported these findings (Figure S9B). Collectively, these results demonstrate that the combination of TPBE scaffolds with mild photothermal stimulation significantly enhances axonal regeneration and NeuN expression in DRG neurons in vitro. These enhancements are likely attributed to the rapid release of Eu^3^^+^/PO_4_^3^^−^ during initial NIR irradiation and the concurrent mild local thermal effect, which collectively enhance neurite outgrowth and differentiation in PC12 cells. Tuj1 is a key component of the early neuronal microtubule skeleton, whereas MAP2 maintains dendritic structure and synaptic plasticity in mature neurons. NeuN serves as a reliable marker for mature neurons and CGRP, which is a sensory neuropeptide, directly supports osteogenic during neuro–bone crosstalk. Downregulation of Nestin expression indicates the exit of neural progenitor cells from a stem state, whereas reduced GFAP expression suggests suppressed astrocyte differentiation [65, 66]. These results show that the prepared TPBE scaffold combined with NIR irradiation effectively induces PC12 cells to differentiate toward mature neurons, involving molecules such as CGRP that regulate neuro–bone interactions, thereby providing a neurobiological foundation for subsequent “neuro–vascular–bone” cascade regeneration during bone repair.

### 3.5 Transcriptomic Analysis Revealing the Potential Mechanism of the PI3K/Akt Signaling Pathway in the Regulation of Early Neural Regeneration Using the NIR‐Responsive TPBE Scaffolds

**FIGURE 6:**
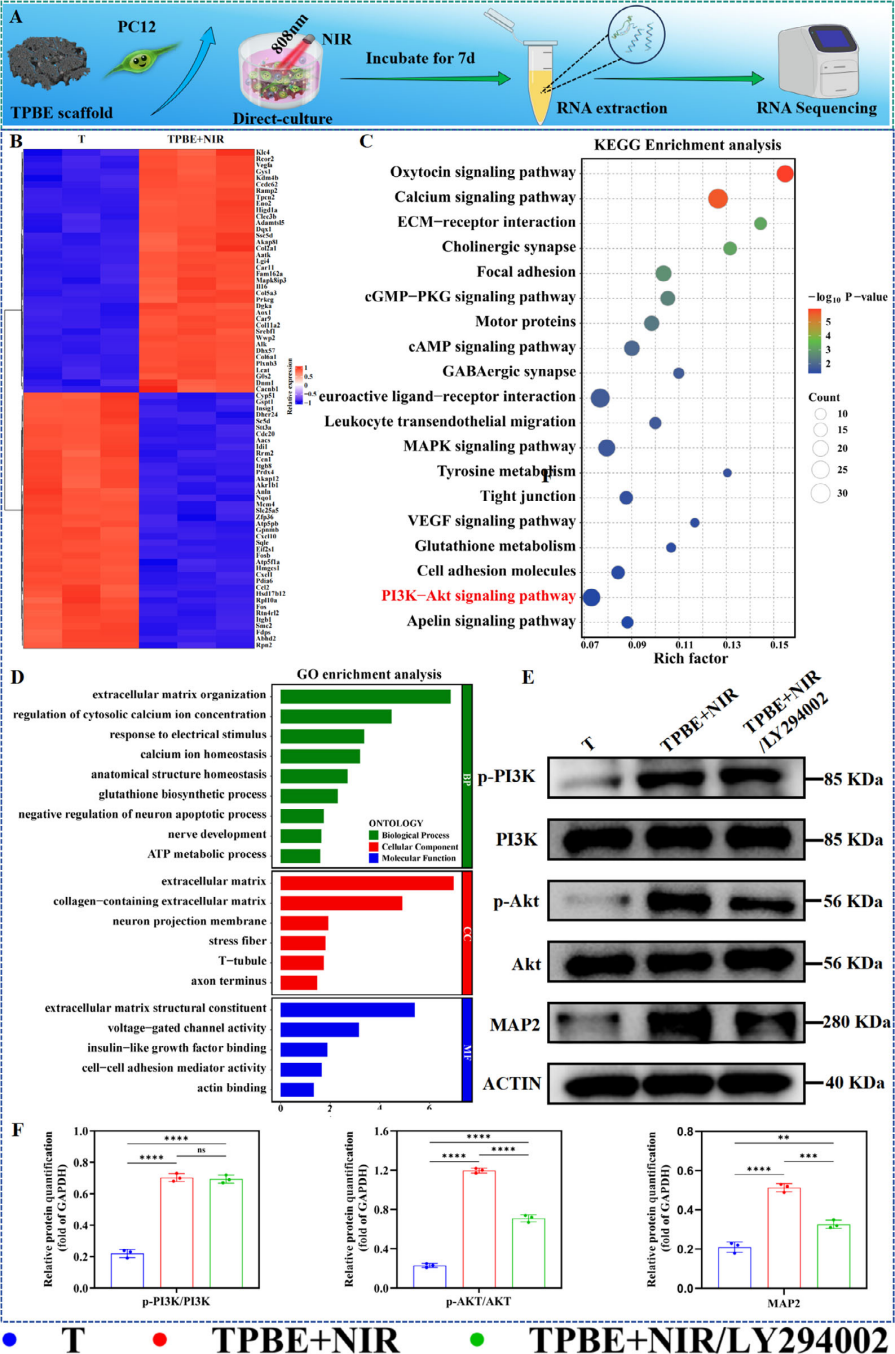
Transcriptomic changes in PC12 cells treated using TPBE‐based NIR stimulation: (A) Schematic of the mRNA sequencing experiments. (B) Heat map visualization of DEGs. (C) KEGG pathway enrichment analysis. (D) GO classification of upregulated DEGs in the TPBE+NIR group. (E,F) Western blot analysis of neuroregeneration‐related proteins (PI3K, p‐PI3K, AKT, p‐ AKT, and MAP2) and quantitative results after 7 days of direct contact. Data are expressed as mean ± SD (*n* ≥ 3). ns: not significant, *p < 0.05, **p < 0.01, ***p < 0.001.

To elucidate how TPBE scaffolds combined with NIR irradiation regulate early neuroactive molecules, mRNA transcriptome sequencing was performed on PC12 cells cultured on TPBE+NIR scaffolds and T (control) scaffolds (Figure 6A). Principal component analysis (PCA) provided an overall assessment of the transcriptome data from both groups. The results revealed considerable intergroup differences, confirming reliable data quality suitable for subsequent in‐depth analysis (Figure S10A). A volcano plot of the RNA‐seq results identified 1443 DEG_S_ in the TPBE+NIR scaffolds relative to the T group, including 928 upregulated and 515 downregulated genes (Figure S10 B). A cluster heatmap (Figure 6B) of differentially expressed genes further confirmed the significant gene expression differences between the two groups of samples. GO analysis functional enrichment analysis indicated that these different genes were considerably enriched in biological processes such as extracellular matrix organization, regulation of cytosolic calcium ion concentration, negative regulation of neuron apoptotic process, adenosine triphosphate (ATP) metabolic process, and nerve development. In addition, cellular components related to neural structure and function, including extracellular matrix and T‐tubule, were considerably enriched (Figure 6D). KEGG pathway analysis further confirmed that differentially expressed genes were significantly enriched in multiple neural regeneration‐related signaling networks, including calcium, MAPK, and PI3K‐Akt signaling (Figure 6C). Notably, the PI3K/Akt pathway is directly involved in regulating neurite formation and neuronal survival, with numerous signals promoting neurite growth converging into this pathway [67, 68]. Thus, this study focused on functional validation of this pathway.

To verify whether TPBE+NIR‐mediated neuronal differentiation and neurite formation depend on the PI3K/Akt signaling pathway, this study used the PI3K‐specific inhibitor LY294002 as an intervention. The experimental groups included the T, TPBE+NIR, and TPBE+NIR+LY294002 groups (all except Group T received NIR irradiation). The protein blot results (Figure 8E,F) demonstrated that, compared to Group T, the ratios of p‐PI3K/PI3K and p‐Akt/Akt were significantly elevated in the TPBE+NIR group, accompanied by a synchronous upregulation of the neuronal differentiation marker MAP2 expression. Upon addition of LY294002, photothermal stimulation‐induced p‐Akt phosphorylation was markedly suppressed, and MAP2 expression was substantially reduced, indicating effective blockade of the PI3K/Akt pathway. Notably, p‐PI3K levels did not decline synchronously, consistent with LY294002's mechanism as a competitive ATP inhibitor targeting the catalytic subunit of p110, rather than regulating subunit phosphorylation [69, 70, 71]. These findings collectively suggest that TPBE scaffold‐induced neuronal differentiation and neurite formation under NIR stimulation rely on the activation of the PI3K/Akt signaling pathway.

### 3.6 Effects of Scaffolds in Each Group on Angiogenesis of In Vitro HUVECs

**FIGURE 7:**
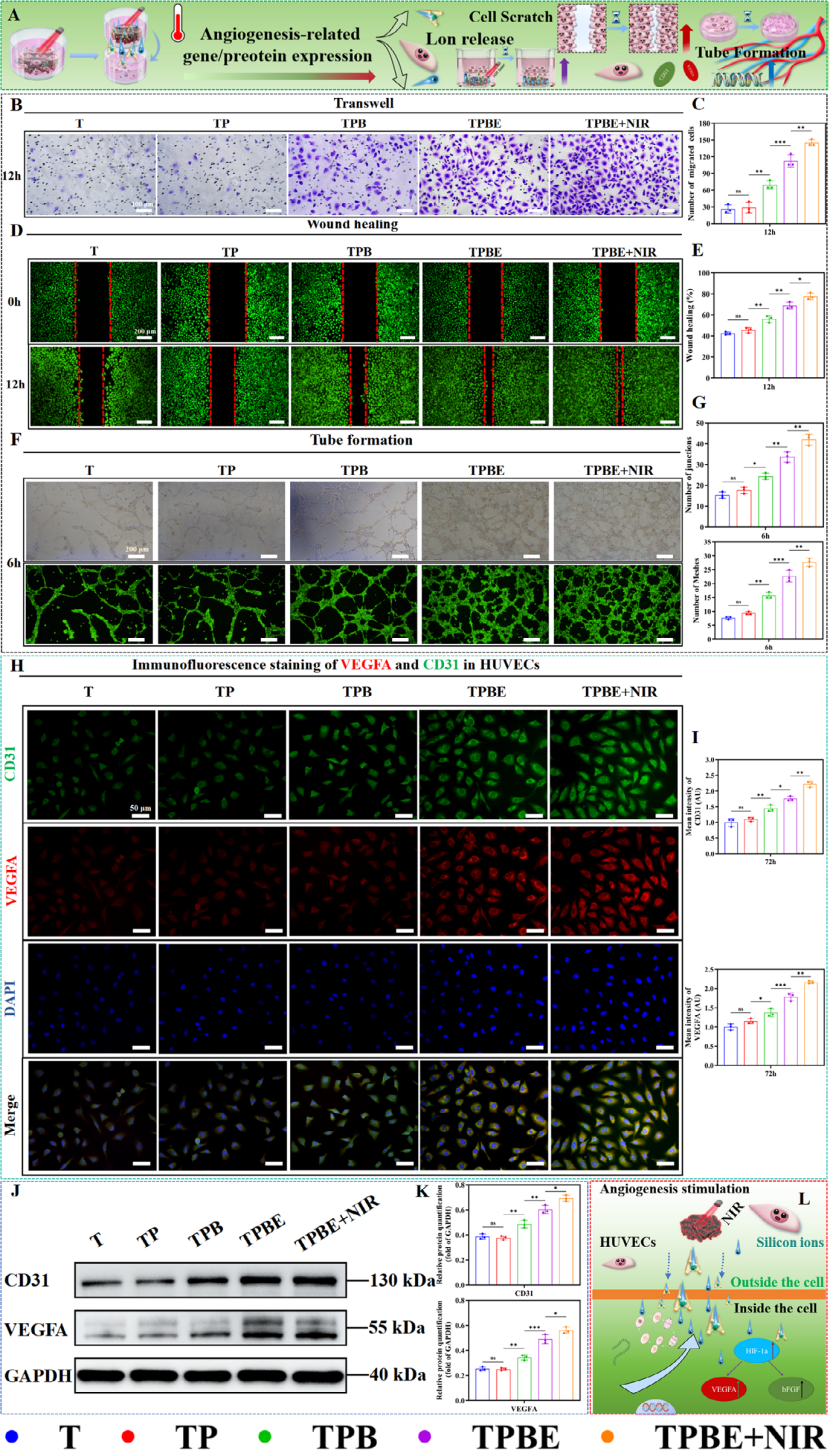
Angiogenic activity of HUVECs cultured on different scaffolds: (A) Schematic of the HUVEC‐scaffold cocultured system under NIR irradiation and vascularization induction. (B,C) Transwell assay images and quantitative analysis (scale bar = 200 µm). (D,E) Scratch wound assay images and migration rate (%) (scale bar = 200 µm). (F,G) Tube formation images and quantitative analysis (scale bar = 200 µm). (H,I) IF staining and quantitative analysis of CD31 and VEGFA after 72 h of indirect coculture (scale bar = 50 µm). (J,K) Western blot analysis of angiogenesis‐related proteins (CD31 and VEGFA) and quantitative results after 72 h of direct contact. (L) Angiogenesis schematic. Data are expressed as mean ± SD (*n* ≥ 3). ns: not significant, *p < 0.05, **p < 0.01, ***p < 0.001.

Promoting angiogenesis is an effective therapeutic approach to accelerate bone defect healing. The formation and maturation of blood vessels provide essential nutrients, oxygen, growth factors, and hormones to regenerate tissues, while offering structural guidance for nerve ingrowth and creating a suitable microenvironment for mineralization [72, 73]. Hence, the angiogenic effects of the TPBE scaffolds were investigated with and without NIR irradiation (Figure 7A). The chemotactic effect of the TPBE scaffolds on HUVECs was evaluated using transwell assays. Compared with T and TP scaffolds, the TPB group recruited more HUVECs to the lower surface of the upper chamber, indicating that the high‐specificity affinity of BPNSs for endothelial cells was effectively preserved (Figure 7B) [74]. This migratory effect was further enhanced under periodic NIR irradiation, with the highest number of migrating cells observed in the TPBE+NIR group (Figure 7C), followed by the TPBE group. Additionally, the in vitro migratory capacity of HUVECs was assessed using scratch wound assays. Both the TPBE and TPBE+NIR groups, particularly the latter, exhibited superior scratch healing at 12 h compared with the other groups (Figure 7D,E). These findings were consistent with transwell migration data, confirming the synergistic pro‐angiogenic effect of PDA@(BP+Eu) nanosheets combined with mild thermal stimulation in the photoactivated TPBE scaffold. During the early stages of tissue regeneration and remodeling, recruitment of vascular endothelial cells to bone defect sites is crucial for subsequent angiogenic differentiation and considerably promotes tissue repair [75]. Hence, the enhanced cell migration and superior angiogenic capacity exhibited by the TPBE+NIR group scaffolds offer a promising strategy for accelerating in vivo bone defect healing.

We employed in vitro tubing assays to further investigate the pro‐angiogenic capacity of the TPBE scaffolds (Figure 7F,G). After 6 h, the T and TP groups formed incomplete or sparse tubular networks. Although the TPB group promoted tubular structure formation, the TPBE+NIR group exhibited a considerably more pronounced effect on vascular tubular formation. The resulting capillary‐like network was more mature than that of the other groups, with the TPBE group ranking second (Figure 7F). This was primarily attributed to the rapid release of pro‐angiogenic Eu^3+^/PO_4_^3−^ active ions from PDA@(BP+Eu) nanosheets within the TPBE scaffold under NIR stimulation. Notably, Eu^3+^ is a key pro‐angiogenic cellular ion that promotes endothelial cell migration, proliferation, and angiogenesis [61]. In addition, a quantitative analysis confirmed that the TPBE+NIR group exhibited considerably number of nodes and network density than all the other groups, followed by the TPBE group (Figure 7G). These results indicate that combining TPBE scaffolds with mild photothermal treatment synergistically enhances the pro‐angiogenic properties of HUVECs.

At the molecular mechanism level, HUVECs were directly cultured using scaffolds from each group for 72 h, whereas the expression of angiogenesis‐related markers was evaluated. The qRT‐PCR (Figure S11) results showed that, compared with the T and TP groups, the TPB group upregulated VEGFA, CD31, and HIF‐1α. This indicates that BPNSs promote angiogenesis by regulating vascular endothelial cell metabolism, enhancing proliferation, and activating angiogenesis marker expression [14]. The TPBE scaffolds exhibited more pronounced gene expression. This enhancement resulted from gradual degradation of PDA coating and BPNSs, which synergistically promoted Eu^3^^+^ release and facilitated efficient vascular network formation [76]. Furthermore, combining TPBE scaffolds with mild photothermal treatment further increased the expression of angiogenesis genes. This improvement may be attributed to NIR irradiation‐induced mild thermotherapy, which accelerates angiogenesis by regulating angiogenic factor expression [54]. The Western blot and IF staining further validated these trends at the protein level. HUVECs cocultured directly with each scaffold for 72 h exhibited considerably higher CD31 and VEGFA expressions in the TPBE+NIR group than the other groups, as confirmed by the results of the gray‐scale and quantitative analyses (*p* < 0.05) (Figure 7J,K). Further, IF staining after 72 h of indirect coculture demonstrated considerably enhanced fluorescence intensity for CD31 and VEGFA in the TPBE+NIR group (Figure 7H,I). Timely and rapid vascularization during bone repair is regulated by VEGFA, CD31, and downstream angiogenesis factors such as hypoxia‐inducible factor 1α (HIF‐1α) [77]. These findings indicate that the TPBE scaffold platform, through the synergistic effects of photothermal‐triggered ion release and local thermo‐microenvironment, co‐upregulated HIF‐1α, which in turn activated the VEGFA/CD31 signaling axis via paracrine mechanisms. This enhanced endothelial cell proliferation, migration, and in vitro, thereby promoting rapid vascular network construction and supporting microenvironmental remodeling for bone regeneration (Figure 7L).

### 3.7 Effects of Scaffolds in Each Group on Osteogenic Differentiation of In Vitro rBMSCs

**FIGURE 8:**
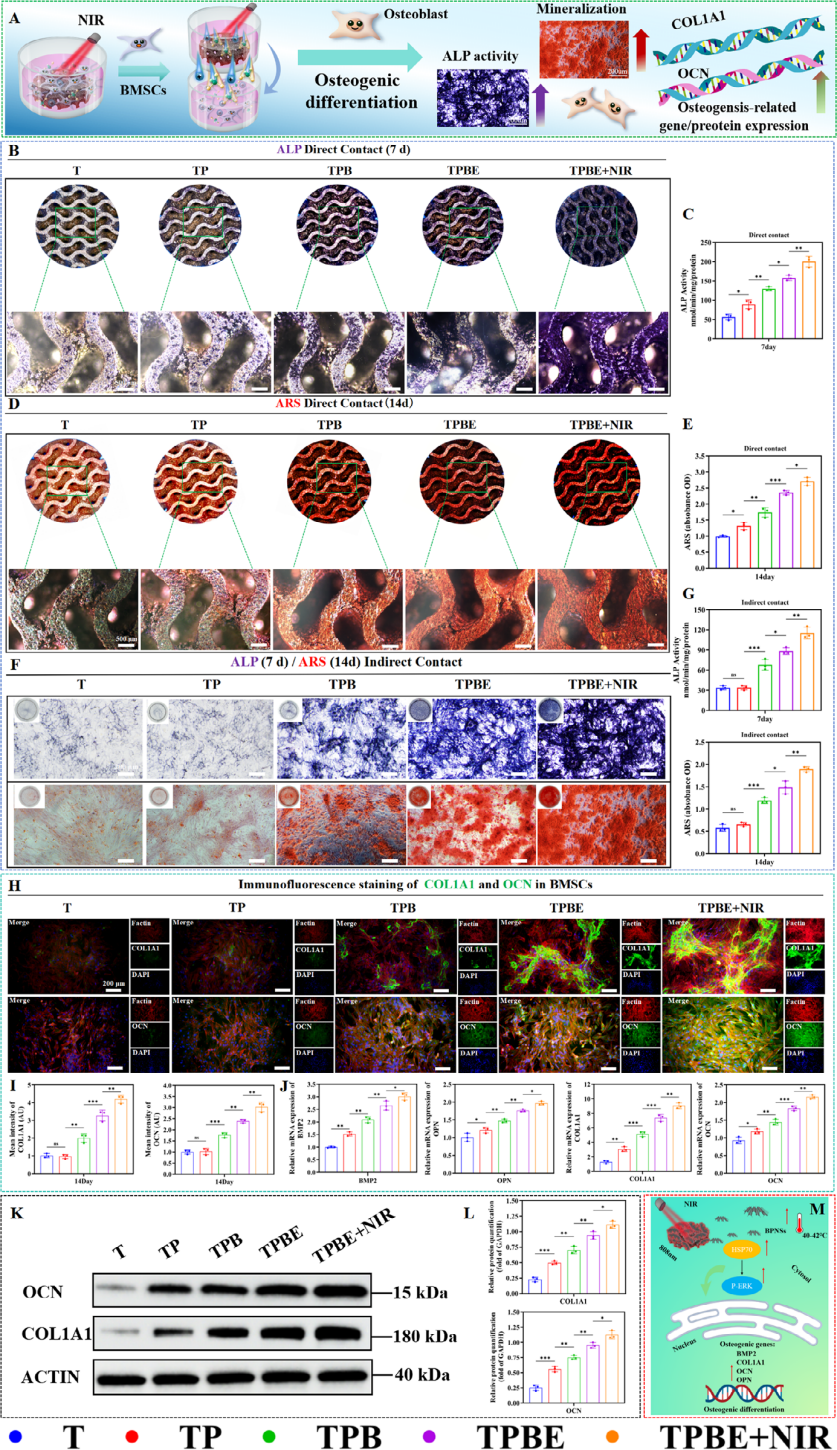
Effects of different scaffolds on the osteogenic differentiation and mineralization of rBMSCs: (A) Schematic of the rBMSC‐scaffold coculture system under NIR irradiation and osteogenic differentiation induction. (B,C) ALP staining after seven days of direct contact coculture with rBMSCs and quantitative analysis (scale bar = 500 µm) (D,E) ARS staining after 14 days of direct contact coculture with rBMSCs and quantitative analysis (scale bar = 500 µm). (F,G) ALP staining after 7 days of indirect contact, ARS staining after 14 days, and quantitative analysis (scale bar = 200 µm). (H,I) IF staining and quantitative analysis of COL1A1 and OCN after 14 days of indirect coculture (scale bar = 200 µm). (J) RT‐qPCR analysis of osteogenesis‐related genes (BMP2, COL1A1, OCN, and OPN) after 14 days of direct contact. (K,L) Western blot analysis of osteogenesis‐related proteins (COL1A1 and OCN) and quantitative results after 14 days of direct contact. (M) Schematic of osteogenesis. Data are expressed as mean ± SD (*n* ≥ 3). ns: not significant, *p < 0.05, **p < 0.01, ***p < 0.001.

In addition to excellent cell compatibility and neurovascularization, robust osteogenic activity is crucial for successful bone regeneration at the scaffold‐host interface. Osteogenic functional progression involves three stages: cell adhesion and proliferation, directed differentiation, and matrix mineralization. These are regulated by the physicochemical cues from the extracellular matrices [78, 79]. We evaluated the regulatory effect of TPBE scaffolds on rBMSCs osteogenic differentiation under NIR irradiation using ALP activity and alizarin red S (ARS) staining (Figure 8A). After seven days of direct coculture, ALP staining revealed higher expression in the TPBE group than in the T and TP groups, with the TPBE+NIR group exhibiting the strongest signal (Figure 8B). A quantitative analysis (Figure 8C) further confirmed that ALP activity was considerably higher in the TPBE+NIR group than in the other groups. Similar trends were observed for matrix mineralization in the 14‐d ARS staining results. As shown in Figure 8D, the T group forms only sparse mineralized nodules, indicating limited osteogenic potential. The TP group demonstrates enhanced mineralization owing to the PDA coating, which is rich in functional groups such as catechol and imine that promote cell differentiation [35]. A TPB treatment further stimulates extracellular mineralized nodule formation, indicating that the PO_4_^3−^ released from BPNSs synergistically interacts with Ca^2+^ to promote in situ formation of hydroxyapatite within the ECM, thereby accelerating osteoblast differentiation [13]. Under periodic NIR irradiation, the TPBE+NIR group exhibits the highest calcium nodule formation and spatial distribution density, followed by the TPBE group. This may be attributed to the Eu^3^^+^ ions released under NIR regulation, which are known to promote osteoblast proliferation and differentiation [53]. A quantitative statistical analysis confirms the superior osteogenic capacity of the TPBE+NIR group (Figure 8E). The results of indirect coculture experiments are consistent with these findings, which demonstrate considerably higher ALP and ARS staining in the TPBE+NIR group compared with the other groups. However, no considerable differences are observed between the T and TP groups (*p* > 0.05) (Figure 8F,G).

To further evaluate osteogenic differentiation, qRT‐PCR was employed after 14 days of direct coculture with rBMSCs to quantify osteogenesis‐related gene expression across all groups. As shown in Figure 8J, the TPBE+NIR group exhibits higher mRNA levels of key osteogenic markers (BMP2, COL1A1, OPN, and OCN) [80] compared with the other groups, consistent with the enhanced ALP activity and ARS staining results. A Western blot analysis (Figure 8K) confirms these findings at the protein level. Gray‐scale quantification further confirms these statistically significant differences (*p* < 0.05; Figure 8L). IF staining after 14 days of indirect coculture between cells and scaffolds confirms these results, demonstrating enhanced fluorescence intensities of COL1A1 and OCN in the TPBE+NIR group (Figure 8H,I).

Recent studies have indicated that mild photothermal stimulation promotes osteoblast recruitment by activating heat shock proteins (HSPs), which regulate matrix metalloproteinase and extracellular signal‐regulated kinase (ERK)–Wnt/β‐catenin–BMP2 signaling pathways [27, 39]. Given the critical role of HSPs in photothermal‐mediated tissue regeneration, RT‐PCR and Western blot analysis were employed to assess heat shock protein 70 (HSP70) expression. As shown in Figure S12A–D, no considerable differences in HSP70 gene and HSP70 protein levels are observed among groups under conventional culture conditions (*p* > 0.05). However, the HSP70 expression considerably increases in the TPBE+NIR group after NIR treatment, coupled with elevated phosphorylated ERK levels. The upregulation of HSP70 is recognized as a crucial mechanism underlying thermal stimulation enhancement of osteogenic differentiation [81]. Hence, these results indicate that PDA@(BP+Eu) nanosheet coatings, combined with NIR‐assisted mild photothermal stimulation, effectively activate HSP70 signaling at elevated temperatures, thereby enabling osteoblast differentiation and mineralization and enhancing the osteoinducive capacity of TPBE scaffolds (Figure 8M). This effect may provide a mechanistic basis for future in vivo bone defect repair applications.

### 3.8 Performance in Reconstructing Neurovascularization Network and Promoting In Vivo Bone Regeneration of Scaffolds

**FIGURE 9:**
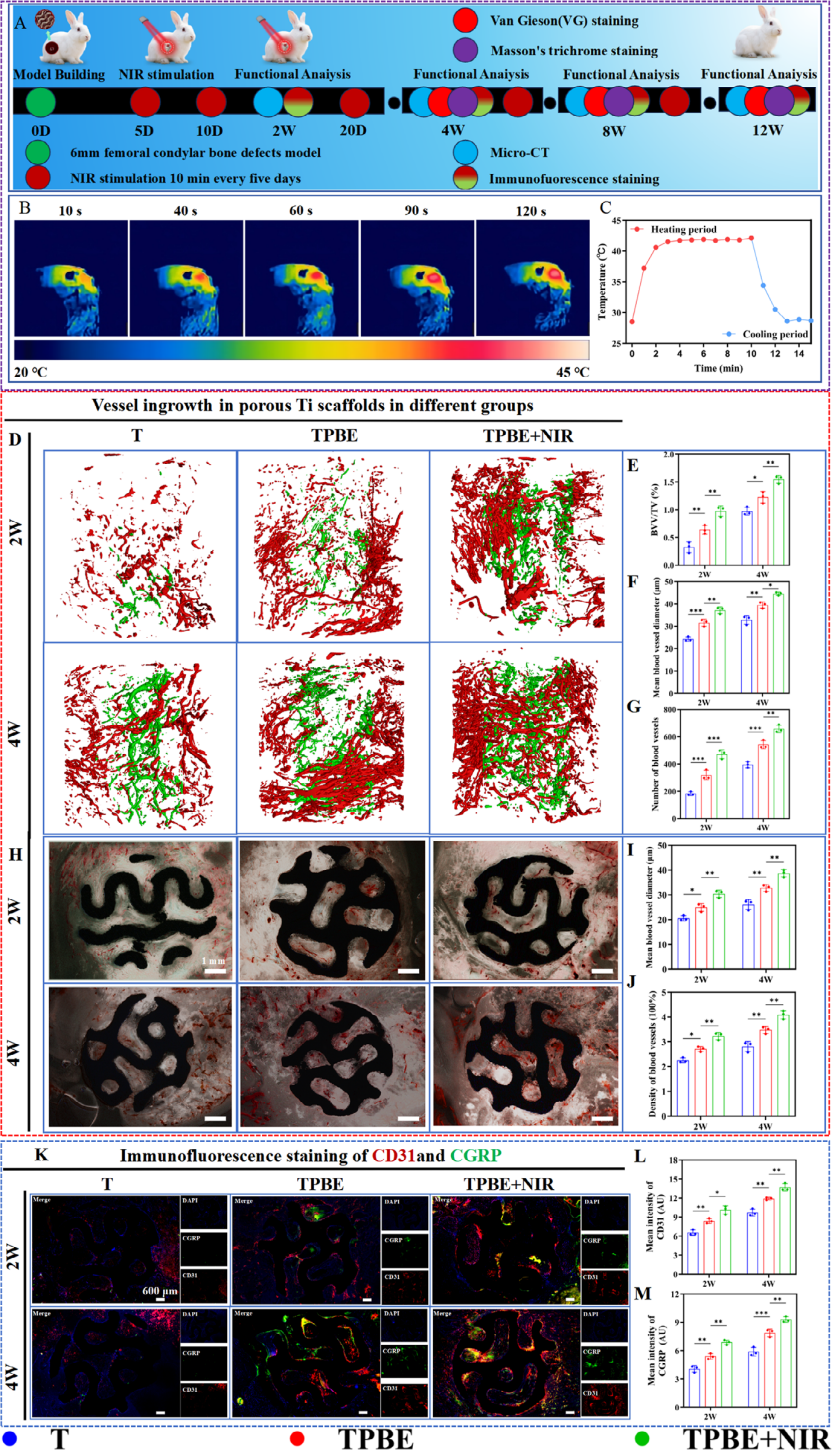
Vascularization within porous titanium scaffolds across different groups: (A) Schematic of the rabbit femoral condyle defect model and experimental workflow. (B) Infrared thermal imaging during in vivo NIR irradiation. (C) In vivo temperature elevation curves of the scaffold. (D) 3D micro‐CT reconstructed images of vascular networks within and around porous titanium scaffolds at 2 and 4 weeks after implantation (green: blood vessels inside the scaffold, red: blood vessels 2 mm outside the scaffold). (E) Quantitative analysis of vascular volume within the scaffolds. (F) Mean vessel diameter within the scaffolds; (G) Number of vascular networks within the scaffolds. (H) Histological images of vascular networks in the scaffold sections (scale bar = 1 mm). (I,J) Quantitative analysis of mean vessel diameter and vascular density. (K) Representative IF images of CD31 (red) and CGRP (green) in the defect region at 2 and 4 weeks after implantation (scale bar = 600 µm). (L,M) Quantitative analysis of CD31 and CGRP fluorescence intensity. Data are expressed as mean ± SD (*n* ≥ 3). ns: not significant, *p < 0.05, **p < 0.01, ***p < 0.001.

To evaluate the therapeutic efficacy of TPBE scaffolds in in vivo bone repair under NIR regulation, a large‐segment femoral condyle defect model (*Φ* = 6 mm) was developed in a rabbit model, followed by periodic NIR irradiation every five days after implantation (Figure 9A). As shown in Figure 9B,C, the TPBE+NIR group receives irradiation at 808 nm (1.0 W/cm^2^) based on the preset protocol. Two minutes after irradiation, the defect‐site temperature increases to 40.6°C and remains at 40–42°C for the next 8 min. After natural cooling, it returns to baseline within 3 min. This rapid thermal recovery indicates that the local photothermal effect is transient and controllable, thereby reducing the risk of local or systemic thermal injury [82].

Previous studies have demonstrated that enhanced vascularization accelerates mesenchymal stromal cell (MSC) recruitment, thereby promoting osteoprogenitor differentiation and mineralization [3]. Hence, micro‐CT was first employed to perform 3D morphological analysis of the scaffold and surrounding 2‐mm ROI and evaluate the ability of the TPBE scaffold to promote early vascular network reconstruction under NIR regulation. Figure 9D shows limited vascular formation in the T group at 2 and 4 weeks after implantation and considerably enhanced angiogenesis in the TPBE group. This effect was further strengthened under cyclic NIR irradiation, indicating superior pro‐angiogenic capacity. A quantitative analysis confirmed that the TPBE and TPBE+NIR groups, particularly the latter, developed more mature and continuous vascular networks, with considerably greater vascular volume, mean diameter, and cross‐sectional area than the T group (*p* < 0.05) (Figure 9E–G). The results from hard tissue sections were consistent with micro‐CT findings. As shown in Figure 9H, the T group exhibits sparse and discontinuous vascular morphology, whereas the TPBE group exhibits distinct vascular clusters within the scaffold pores as early as week 2, with increasingly denser vascular distribution by week 4. After periodic NIR irradiation, vascular regeneration within and around the TPBE scaffold becomes more pronounced. A quantitative analysis further demonstrates larger mean vessel diameter and vascular density in the TPBE group than in the T group. These pro‐angiogenic effects are further enhanced by mild photothermal treatment (Figure 9I,J).

Because of the critical role of neurovascular networks in bone repair, IF staining was performed on tissue sections from each group to assess angiogenesis and neurogenesis. As shown in Figure 9K, CD31 (red) and CGRP (green) expressions in the TPBE and TPBE+NIR groups increase considerably at 2 and 4 weeks after implantation than in the T group, indicating enhanced host neurovascular infiltration. Pronounced colocalization of CD31 and CGRP is observed in the TPBE+NIR group, consistent with the in vitro upregulation of the VEGFA/CGRP signaling pathway and enhanced neural differentiation of PC12 cells. These findings indicate that the bioactive microenvironment created by the scaffold enables early in vitro and in vivo neurovascular coupling [83, 84]. A quantitative fluorescence intensity analysis further confirms this trend, with the CD31 and CGRP expressions highest in the TPBE+NIR group, followed by the TPBE and T groups (Figure 9L,M). CD31 serves as a specific endothelial cell marker [85], whereas CGRP, which is primarily produced by neurons, promotes osteoblast differentiation by activating CGRP receptors [86]. These findings demonstrate the superior angiogenic and neurogenic potential in the TPBE+NIR group.

**FIGURE 10:**
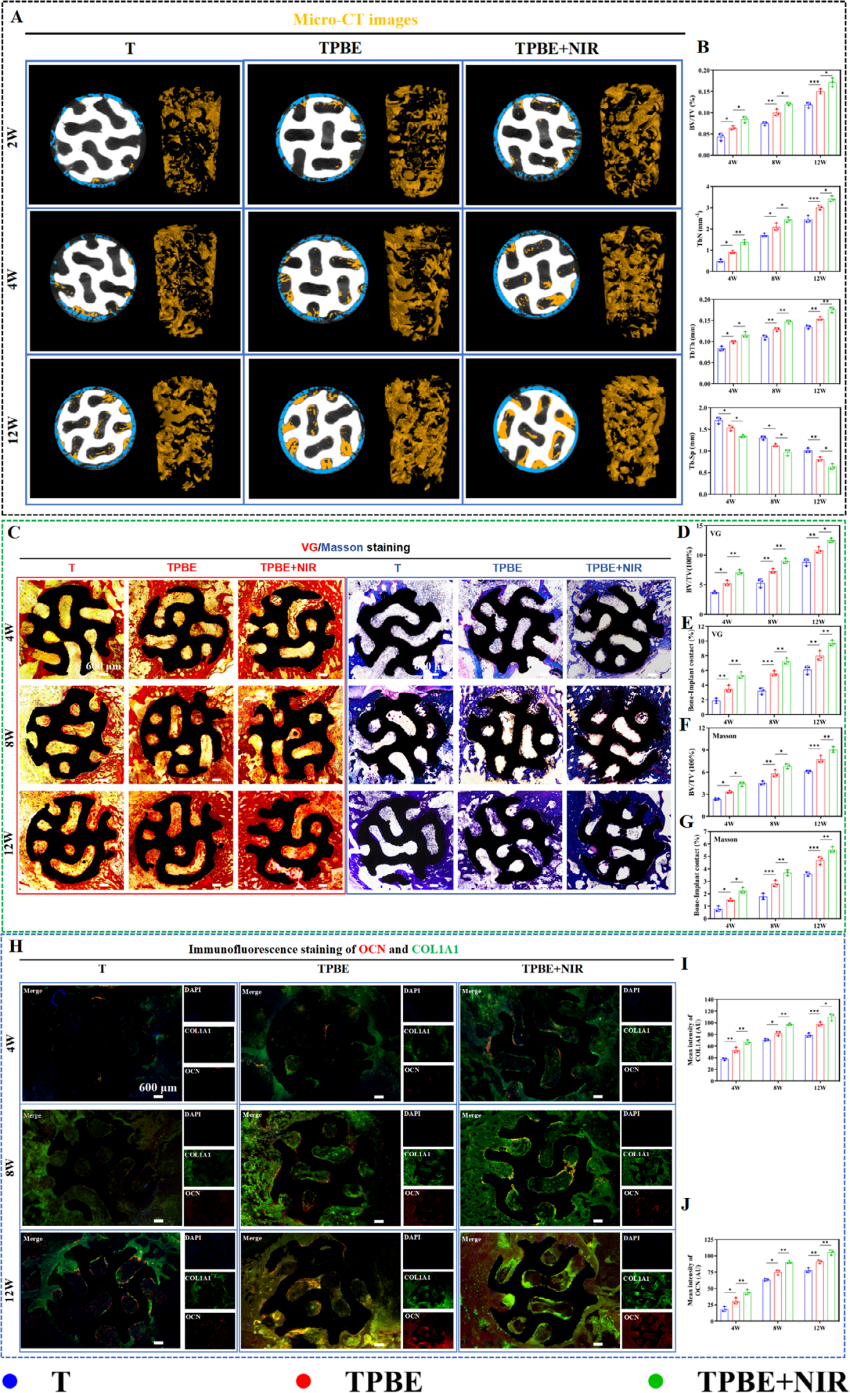
Osteointegration effects of different porous titanium scaffolds: (A) Micro‐CT images showing bone ingrowth within the scaffolds at 4, 8, and 12 weeks after implantation (yellow regions indicate new bone tissue). (B) Quantitative analyses of bone volume fraction (BV/TV), trabecular number (Tb.N), trabecular thickness (Tb.Th), and trabecular separation (Tb.Sp). (C) Representative VG and Masson's trichrome‐stained histological sections of the implant site (scale bar = 600 µm). (D,E) Quantitative analyses of BV/TV and BIC of the representative VG stain. (F,G) Quantitative analyses of BV/TV and BIC of the representative Masson's trichrome stain. (H) Representative IF images of newly formed OCN (red) and COL1A1 (green) in the defect area after implantation for the 4‐, 8‐, and 12‐week group (scale bar = 600 µm). (I,J) Quantitative analyses of OCN and COL1A1 fluorescence intensities. Data are expressed as mean ± SD (*n* ≥ 3). ns: not significant, *p < 0.05, **p < 0.01, ***p < 0.001.

To quantify the repair efficacy of defective bone tissue, the regenerative performance of the TPBE scaffolds was further evaluated. Micro‐CT imaging (Figure 10A) showed minimal new bone formation during healing in the T group at three different time points, indicating poor bone regenerative ability. At 12 weeks after implantation, the TPBE+NIR and TPBE scaffolds demonstrated excellent osteogenic induction during healing, which was characterized by a new bone tissue extending from the defect margins toward the center more than the T group, with the TPBE+NIR group achieving near‐complete defect repair and continuous, mature bone tissue formation within the scaffold. A quantitative morphometry analysis (Figure 10B) further confirmed considerably higher bone volume fraction (BV/TV), trabecular thickness (Tb.Th), and trabecular number (Tb.N), and lower trabecular separation (Tb.Sp) in the TPBE‐treated groups than in the T group (*p* < 0.05), with the TPBE+NIR group exhibiting the highest favorable values. Specialized histological staining (VG and Masson) of hard tissue sections indicated that the TPBE+NIR group exhibited the most pronounced collagen deposition and osteogenic activity at all time points (Figure 10C,D,F). Furthermore, its BIC rate was considerably higher than that of the T group, followed by the TPBE group (Figure 10E,G). Qualitative and quantitative IF staining analyses further revealed that, as the repair progressed, the TPBE+NIR group exhibited the strongest OCN and COL1A1 expressions, followed by the TPBE group, whereas the T group exhibited the lowest levels (Figure 10H–J). These findings indicate enhanced scaffold bone matrix synthesis, mineralization, and metabolic remodeling in the TPBE+NIR group [87]. Furthermore, HE staining (Figure S13B) and serum biochemical analyses (Figure S13A) of major organs (heart, liver, spleen, lung, and kidney) after 12 weeks of implantation confirmed the excellent biosafety of TPBE scaffolds under NIR irradiation, with no detectable systemic toxic.

The results confirm that TPBE scaffolds, in conjunction with mild photothermal effects, effectively activate the PI3K/Akt signaling cascade, thereby promoting early neuronal differentiation and neurite growth. Short‐term (12 W) in vivo implantation studies revealed no significant pathological damage to major organs, providing preliminary evidence of the material's excellent biocompatibility. The system provides a reliable research platform for establishing a neuro–vascular–bone coupled regulatory signaling network. However, several limitations remain. Pharmacological inhibitor interventions suggest that the PI3K/Akt pathway is associated with regulatory mechanisms; however, they cannot fully elucidate the complex causal relationships involved. Future studies should employ PI3K gene overexpression, knock‐in/knock‐out cell models, and animal models combined with primary DRG cell systems to further clarify its regulatory logic. Additionally, long‐term in vivo degradation kinetics of the PDA@(BP+Eu) coating and the potential accumulation of PO_4_^3−^ and Eu^3+^ in tissues remain unclear, necessitating systematic long‐term in vivo investigations to establish a solid theoretical and experimental bases for the clinical translation of such functional bone repair materials.

## 4 Conclusion

We developed a NIR‐responsive smart scaffold system using PDA@(BP+Eu) bioactive nanosheets as the core functional unit. The system can effectively address the low bone regeneration efficiency and poor long‐term stability of conventional titanium implants, which result from the lack of neurovascular regeneration‐inducing activity on the implant surface. By addressing these limitations, the scaffold enables sequential cascade regulation of neurovascularization and osteogenic differentiation during bone repair. This “neurovascular‐led, osteogenic‐targeted” cascade mechanism establishes a microsystem foundation for efficient bone regeneration. Its potential mechanisms are summarized as follows: (1) the 3D porous TPMS titanium scaffold mimics the natural bone trabeculae, providing an ideal mechanical microenvironment that supports cell adhesion, growth, migration, and differentiation, thereby accelerating bone healing; (2) elevated photothermal treatment temperatures trigger controlled initial burst release of Eu^3^^+^/PO_4_^3−^ from PDA@(BP+Eu) nanosheets on the TPBE scaffold, enabling rapid neurovascular network formation during early repair; (3) the combination of TPBE scaffolds with mild photothermal effects promotes neuronal differentiation and neurite formation, which depends on the activation of the PI3K/Akt signaling pathway. These findings demonstrate that TPMS scaffolds, functionalized with PDA@(BP+Eu) nanosheets and combined with mild photothermal treatment, offer a promising strategy for promoting bone regeneration in large defects by sequentially enhancing early neurovascularization and sustained osteogenesis.

## Author Contributions

**Bingqian Wang**: writing – review & editing, writing – original draft, methodology, investigation, funding acquisition, formal analysis, data curation, conceptualization. **Yuping Zhang: **software, methodology, investigation, formal analysis, data curation, conceptualization. **Xiaoning Su**: visualization, software, methodology.** Ning Liu**: methodology, investigation, formal analysis, data curation, conceptualization. **Hui Zeng: **software, methodology, formal analysis, data curation. **Bo Yang**: Software, Formal analysis. **Yang Xue**: review & editing, Supervision. **Fang Guo**: writing – review & editing, resources, project administration, methodology, funding acquisition, conceptualization. **Changkui Liu**: writing – review & editing, supervision, resources, methodology, investigation, funding acquisition.

## Ethics Statement

Experimental and animal care procedures were approved by the Xi'an Medical University Ethics Committee (Number: XYLS2024120).

## Conflicts of Interest

The authors do not have any possible conflicts of interest, and declare that they have no known competing financial interests or personal relationships that could have appeared to influence the work reported in this paper.

## Supporting information




**Supporting File**: advs77652‐sup‐0001‐SuppMat.docx.

## Data Availability

All data supporting the main findings of this study are provided in the article. Raw sequencing data are available upon request from the corresponding author.
