# Supplementary material for: Sequential Regulation of Neurovascular Bone Regeneration in Large Bone Defects Using Black Phosphorus‐Europium Nanosheet Integrated TPMS Biomimetic Scaffold with Photo‐Controlled Ion Release
Source: Adv Sci (Weinh). 2026 Sep 16:e77652. Online ahead of print. doi: 10.1002/advs.77652 (PMC13580173; doi:10.1002/advs.77652)
Supplement: Supplementary file 1 [file ADVS-9999-e77652-s001.docx]

Supporting Information

**Sequential regulation of neuro–vascular–bone regeneration in large bone defects using black-phosphorus–europium nanosheet-integrated TPMS biomimetic scaffold with photo-controlled ion release**

**Bingqian Wang^1^, Yuping Zhang^1^, Xiaoning Su^1^, Ning Liu^1^, Hui Zeng^1^, Bo Yang^1^, Yang Xue^2^, Fang Guo^1**^, Changkui Liu^1*^**

*^1^Engineering Research Center Oral biomaterials and advanced equipments, Research Center of Dental and Maxillofacial Tissue Regeneration andRepair Technology, School of Stomatology, Xi'an Medical University, Xi'an, 710021, China*

*^2^State Key Laboratory of Oral and Maxillofacial Reconstruction and Regeneration, National Clinical Research Center for Oral Diseases, Shaanxi Clinical Research Center for Oral Diseases, Department of Oral and Maxillofacial Surgery, School of Stomatology, The Fourth Military Medical University, Xi'an, 710021, China*

*Corresponding author. Changkui Liu Email: liuchangkui@xiyi.edu.cn

Fang Guo Email: guofang@xiyi.edu.cn

**Table S1. Primers used in Real-Time PCR for neurogenesis-related genes.**

| **Gene** | **Forward primer sequence(5’-3')** | **Reverse primer sequence(5’-3')** |
| --- | --- | --- |
| **Tuj1** | CAACTATGTGGGGGACTCGG | TGGCTCTGGGCACATACTTG |
| **MAP2** | GAGAAGGAGGCCCAACACAA | TGTTTGTTCTGATGCTGGCG |
| **CGRP** | GGCCACTCTCAGTGAAGAAGA | TCTGGGCAGTGACACTAGAGC |
| **GFRP** | TTGACCTGCGACCTTGAGTC | GAGTGCCTCCTGGTAACTCG |
| **Nestin** | GGTAGGGCTAGAGGACCCAA | TGGGCAATTCAAGGATCCCC |
| **GAPDH** | CAAGTTCAACGGCACAGTCAAGG | ACATACTCAGCACCAGCATCACC |

**Table S2. Primers used in Real-Time PCR for angiogenesis-related genes.**

| **Gene** | **Forward primer sequence(5’-3')** | **Reverse primer sequence(5’-3')** |
| --- | --- | --- |
| **CD31** | TCGTGGTCAACATAACAGAACT | TTGAGTCTGTGACACAATCGTA |
| **VEGFA** | ATCGAGTACATCTTCAAGCCAT | GTGAGGTTTGATCCGCATAATC |
| **HIF1a** | GAACGTCGAAAAGAAAAGTCTCG | CCTTATCAAGATGCGAACTCACA |
| **GAPDH** | CACCCACTCCTCCACCTTTGAC | GTCCACCACCCTGTTGCTGTAG |

**Table S3. Primers used in Real-Time PCR for osteogenesis-related genes.**

| **Gene** | **Forward primer sequence(5’-3')** | **Reverse primer sequence(5’-3')** |
| --- | --- | --- |
| **OCN** | GGACCCTCTCTCTGCTCACTCTG | ACCTTACTGCCCTCCTGCTTGG |
| **OPN** | GACGATGATGACGACGACGATGAC | GTGTGCTGGCAGTGAAGGACTC |
| **BMP2** | AAGCGTCAAGCCAAACACAAACAG | CCAGTCATTCCACCCCACATCAC |
| **COL1A1** | TGTTGGTCCTGCTGGCAAGAATG | GTCACCTTGTTCGCCTGTCTCAC |
| **HSP70** | AACTACAAGGGCGAGAACCG | GAGTCGTTGAAGTAGGCGGG |
| **GAPDH** | CAAGTTCAACGGCACAGTCAAGG | ACATACTCAGCACCAGCATCACC |

Figure S1. TEM image and elemental mapping of BPNSs (scale bar=100 nm).

Figure S2. Loading capacity of BPs-Eu with different feeding ratio of Eu/BPNSs.

Figure S3. Taking photos with a digital camera of BPNSs, BP+Eu, and PDA@(BP+Eu) dispersed in PBS exposed to air for 0, 4 and 28 days. Data are expressed as mean ± SD (n≥3).

Figure S4. Stress-strain curve of the TPMS structural scaffold.

Figure S5. Stability analysis of the surface coating on TPBE samples before sterilization, after sterilization, and after five photothermal cycles. (A) SEM and EDS elemental mapping images of the samples in all three groups (scale bar = 100 μm, 1 μm, 300 nm, and 1 mm); (B) Maximum surface temperatures of the samples in the three groups under 10-min irradiation; (C) Raman spectra of the samples in all three groups. Data are expressed as mean ± SD (n≥3).

Figure S6. Infrared thermal images of the TPBE scaffold under 808 nm NIR laser irradiation (1 W/cm^2^). Note:The color bar on the right side of the thermal image indicates the temperature range (unit: 20–50 °C).

Figure S7. (A) Evaluation of the levels of the intracellular ROS in three cell during co-culture with each sample group using DCFH-DA (green fluorescence) (scale bar = 200 µm); (B) Quantitative analysis of fluorescence intensity.Data are expressed as mean ± SD (n≥3). ns: not significant, **p*<0.05,***p* < 0.01,****p* < 0.001，*****p* < 0.0001.

Figure S8.（A）Total neurite length of each cell; (B) Number of branching points each cell; (C) Average neurite length of each cell. Data are expressed as mean ± SD (n≥3). ns: not significant, **p*<0.05,***p* < 0.01,****p* < 0.001.

Figure S9. (A) IF staining and quantitative analysis of Tuj1 and NeuN in primary DRG neurons co-cultured with various types of scaffolds for seven days (scale bar = 100 µm); (B) Mean density of Tuj1 and Maximum neurite length, percentage of carrying neurite cells, total neurite length, number of branching points, and average neurite length; (C) Count of NeuN-positive cells and mean density.Data are expressed as mean ± SD (n≥3).ns: not significant, **p*<0.05,***p* < 0.01,****p* < 0.001.

Figure S10.(A) PCA illustrating the variance between T and TPBE+ NIR groups; (B)Volcano plot of DEGs.

Figure S11.RT-qPCR analysis of angiogenesis-related genes (HIF-1α, CD31, and VEGFA) after 72 h of direct contact.Data are expressed as mean ± SD (n≥3). ns: not significant, **p*<0.05,***p* < 0.01,****p* < 0.001.

Figure S12.(A) mRNA expression of HSP70 after different scaffold treatments; (B–D) Western blot and quantitative analyses of HSP70 and p-ERK expression after 14 d of treatment. Data are expressed as mean ± SD (n≥3). **p*<0.05,***p* < 0.01,****p* < 0.001.

Figure S13.(A) Serum biochemical analysis of the TPBE+NIR and T groups 12 weeks after post-implantation; (B) Hematoxylin and eosin (H&E) staining of tissue sections from the TPBE+NIR and T groups 12 weeks post-implantation (scale bar = 200 µm). Data are expressed as mean ± SD (n≥3).
